# Photoelectrochemical Water‐Splitting Using CuO‐Based Electrodes for Hydrogen Production: A Review

**DOI:** 10.1002/adma.202007285

**Published:** 2021-06-12

**Authors:** Roozbeh Siavash Moakhar, Seyed Morteza Hosseini‐Hosseinabad, Saeid Masudy‐Panah, Ashkan Seza, Mahsa Jalali, Hesam Fallah‐Arani, Fatemeh Dabir, Somayeh Gholipour, Yaser Abdi, Mohiedin Bagheri‐Hariri, Nastaran Riahi‐Noori, Yee‐Fun Lim, Anders Hagfeldt, Michael Saliba

**Affiliations:** ^1^ Department of Bioengineering McGill University Montreal QC H3A 0E9 Canada; ^2^ Non‐Metallic Materials Research Group Niroo Research Institute (NRI) Tehran 14686‐13113 Iran; ^3^ Department of Materials Science and Engineering Sharif University of Technology Azadi Ave Tehran 11155‐9466 Iran; ^4^ Electrical and Computer Engineering National University of Singapore Singapore 119260 Singapore; ^5^ Low Energy Electronic Systems (LEES) Singapore‐MIT Alliance for Research and Technology (SMART) Centre Singapore 38602 Singapore; ^6^ Nanophysics Research Laboratory Department of Physics University of Tehran Tehran 14395‐547 Iran; ^7^ Institute for Corrosion and Multiphase flow Technology Department of Chemical and Biomedical Engineering Ohio University Athens OH 45701 USA; ^8^ Institute of Materials Research and Engineering Agency for Science Technology and Research (A*STAR) 2 Fusionopolis Way, Innovis, #08‐03 Singapore 138634 Singapore; ^9^ Laboratory of Photomolecular Science Ecole Polytechnique Fédérale de Lausanne EPFL SB‐ISIC‐LSPM, Station 6 Lausanne 1015 Switzerland; ^10^ Institute for Photovoltaics University of Stuttgart Pfaffenwaldring 47 D‐70569 Stuttgart Germany; ^11^ Helmholtz Young Investigator Group FRONTRUNNER IEK5‐Photovoltaik Forschungszentrum D‐52425 Jülich Germany

**Keywords:** cupric oxide (CuO), heterojunctions, hydrogen evolution, photocurrent density, photoelectrochemical water splitting

## Abstract

The cost‐effective, robust, and efficient electrocatalysts for photoelectrochemical (PEC) water‐splitting has been extensively studied over the past decade to address a solution for the energy crisis. The interesting physicochemical properties of CuO have introduced this promising photocathodic material among the few photocatalysts with a narrow bandgap. This photocatalyst has a high activity for the PEC hydrogen evolution reaction (HER) under simulated sunlight irradiation. Here, the recent advancements of CuO‐based photoelectrodes, including undoped CuO, doped CuO, and CuO composites, in the PEC water‐splitting field, are comprehensively studied. Moreover, the synthesis methods, characterization, and fundamental factors of each classification are discussed in detail. Apart from the exclusive characteristics of CuO‐based photoelectrodes, the PEC properties of CuO/2D materials, as groups of the growing nanocomposites in photocurrent‐generating devices, are discussed in separate sections. Regarding the particular attention paid to the CuO heterostructure photocathodes, the PEC water splitting application is reviewed and the properties of each group such as electronic structures, defects, bandgap, and hierarchical structures are critically assessed.

## Graphical abstract

The photoelectrochemical water‐splitting method using CuO‐based materials as high‐efficiency photoelectrodes has potential to solve the global energy and environmental problems simultaneously. The usage of appropriate synthesis methods, modifying the CuO crystal structure and creating suitable conditions to increase the specific surface area can dramatically separate the electron–hole pairs and improve the photocurrent density of photoelectrodes.

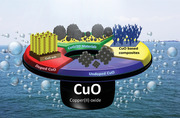

## 1 Introduction

Over the past years, numerous studies have been conducted on developing renewable energies and clean procedures to guarantee a safe and promising future for the planet. Up to now, several strategies have been examined to employ renewable sources of energy such as solar radiation in the solar cells or solar‐driven hydrogen production units.^[^ ^1^, ^2^, ^3^, ^4^, ^5^, ^6^, ^7^, ^8^, ^9^, ^10^ ^]^

Photoelectrochemical (PEC) water splitting is one of the promising procedures to generate hydrogen as a zero‐emission energy carrier alternative to polluting fossil fuels.^[^ ^11^, ^12^, ^13^, ^14^, ^15^, ^16^, ^17^, ^18^, ^19^, ^20^ ^]^ During the PEC water splitting, solar energy can facilitate the occurrence of the hydrogen production half‐reactions at an electrode coated by specialized semiconductors or PEC materials.^[^ ^21^, ^22^, ^23^, ^24^, ^25^, ^26^, ^27^, ^28^ ^]^

To become commercially competitive with other hydrogen production methods, several issues need to be addressed concerning the performance of semiconductor materials.^[^ ^29^, ^30^, ^31^, ^32^, ^33^ ^]^ To begin with, the high solar‐to‐hydrogen (STH) efficiency can be achieved by incorporating narrow‐bandgap semiconductors with high ability of visible light harvesting, enhanced charge carrier (electron–hole) separation, and suitable bandgap position for performing water splitting half oxidation/reduction reactions.^[^ ^34^, ^35^, ^36^, ^37^, ^38^, ^39^ ^]^ Moreover, developing stable photoelectrodes with high resistance against photo‐corrosion is an important feature that should be taken into account. In addition to the physical properties of suitable semiconductors, developing inexpensive materials and electrodes is a major prerequisite for PEC water splitting.^[^ ^40^, ^41^, ^42^, ^43^, ^44^, ^45^, ^46^ ^]^

Copper(II) oxide or cupric oxide is an inorganic nontoxic p‐type semiconductor with the formula of CuO and the ideal narrow bandgap of 1.2–1.7 eV. The strong visible light absorption ability of this oxide makes it a highly promising material for solar water splitting compared to other photoactive metal oxides.^[^ ^47^, ^48^, ^49^, ^50^, ^51^, ^52^ ^]^ The CuO with a narrow bandgap is one of the few photocatalysts with high activity for photocatalysis of HER under simulated sunlight irradiation, especially when combined with other photocatalysts.^[^ ^35^, ^53^, ^54^, ^55^, ^56^, ^57^ ^]^ According to the theoretical investigation, achieving a maximum photocurrent density of 35 mA cm^−2^ has been predicted for the CuO‐based photocathodes.^[^ ^58^ ^]^ Cupric oxide is one of the low‐cost semiconductors that can be obtained by the high amount of copper at earth crust or from the recovery operation of electronic wastes that contain a large amount of Cu wires scraps.^[^ ^59^, ^60^, ^61^, ^62^, ^63^, ^64^ ^]^ Even though cupric oxide is considered a promising ceramic oxide electrode for solar water splitting due to its narrow bandgap, it suffers from some critical drawbacks such as low photostability or photoinduced decomposition that prevent its use as a photocathode in PEC cells. Since the decomposition potential of copper oxide lies close to or within the bandgap, the CuO and Cu_2_O photoelectrodes are prone to photo‐corrosion. In other words, photogenerated electron–hole can participate in the reduction of CuO rather than being involved in the hydrogen production half‐reaction.^[^ ^65^, ^66^, ^67^, ^68^, ^69^ ^]^

The CuO particles or thin films can be prepared via various procedures such as electrodeposition,^[^ ^70^, ^71^ ^]^ spray pyrolysis,^[^ ^72^ ^]^ sol–gel,^[^ ^73^, ^74^, ^75^ ^]^ microwave irradiation synthesis,^[^ ^76^, ^77^, ^78^, ^79^, ^80^, ^81^, ^82^ ^]^ sonochemical,^[^ ^83^, ^84^, ^85^ ^]^ template‐assisted,^[^ ^86^, ^87^, ^88^ ^]^ hydrothermal,^[^ ^89^, ^90^, ^91^ ^]^ thermal oxidation,^[^ ^92^, ^93^, ^94^ ^]^ chemical vapor deposition (CVD),^[^ ^95^ ^]^ and sputtering method.^[^ ^96^, ^97^, ^98^, ^99^, ^100^ ^]^ Each methods has its influences on the direct bandgap and surface characteristics of prepared CuO photoelectrodes such as the size of the particles, specific surface area, and surface morphology.^[^ ^101^ ^]^ These fabrication processes provide a situation of CuO doping with different elements or a combination of CuO with other promising materials that result into the superior development of photocathode.

Although pristine CuO has outstanding features and has shown a promising behavior under simulated sunlight irradiation, challenges of improving the stability of CuO and reducing the photogenerated electron–hole pair recombination still have remained unresolved. These drawbacks reduce the photocatalytic activity of this semiconductor and subsequently prevent the mass production of this material.^[^ ^67^, ^96^, ^102^, ^103^ ^]^

Some of the solutions presented in previous reports, such as controlling the morphology,^[^ ^104^, ^105^, ^106^, ^107^, ^108^, ^109^, ^110^ ^]^ doping, or modification with elements,^[^ ^111^, ^112^, ^113^, ^114^, ^115^ ^]^ and the formation of a heterojunction,^[^ ^116^, ^117^, ^118^, ^119^, ^120^, ^121^, ^122^, ^123^ ^]^ are considered as appropriate approaches to promote the photocatalytic activity of CuO. The effects of some of them are described in the following sections. These practical strategies to some extent can help overcome the restrictions of CuO and improve its photocatalytic performance by constituting one or more properties. Some of these properties are developing the electronic structure of CuO, increasing the specific surface area, reducing the activation energy, enhancing the charge separation ability, and creating new mechanisms for the transmission of electron–hole pairs with bandgap engineering.^[^ ^96^, ^124^, ^125^, ^126^, ^127^, ^128^, ^129^, ^130^ ^]^ In this regard, one of the important strategies for improving the photocatalytic performance of semiconductors is constructing heterogeneous structures. Among the different groups of materials, 2D materials have a distinct feature such that they have experienced significant advances in energy, optic, electronic, and catalytic properties owing to their tunable electronic structure and bandgap, additional internal electric field, and large surface area.^[^ ^131^, ^132^, ^133^ ^]^ Also, their combinations with various semiconductors have become a research hotspot in recent years.^[^ ^134^, ^135^, ^136^, ^137^, ^138^, ^139^, ^140^, ^141^ ^]^ In this regard, particular attention has been paid to CuO/2D materials in this review.

**Figure 1:**
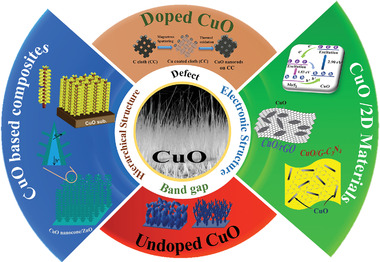
Schematic representation of the topics covered in this review.

To the best of the authors’ knowledge, this is the first comprehensive literature review that covers the PEC water splitting of CuO‐based electrodes for hydrogen production aiming to focus on PEC properties of pure CuO and CuO composites, incorporating metallic elements into the CuO structure (e.g., Ti and Pd), heterojunction formation of CuO with oxide semiconductors (e.g., Cu_2_O, ZnO, and WO_3_), and CuO/2D materials heterojunctions (e.g., 2D carbon material, graphitic carbon nitride (g‐C_3_N_4_), dichalcogenides), and their recent developments. The overview of the topics covered in this review is depicted in **Figure** **1**.

## 2 Overview of Using Metal‐Oxide‐Based Electrodes in PEC Water Splitting

The photocathode and photoanode are designed to convert solar energy to hydrogen efficiently. To reach this goal, several demands and prerequisites should be addressed. To begin with, semiconductor materials should have low bandgap energy for better light‐harvesting in the visible region.^[^ ^142^, ^143^, ^144^, ^145^ ^]^ To date, diverse research has been conducted on materials with a small bandgap (less than 2 eV) and the highest STH efficiencies have been observed in the III–V systems. These systems are those semiconductor materials composed of elements in group IIIA and VA. For instance, the STH efficiency of 12.4% is reported for a tandem configuration containing p‐GaInP_2_ PEC junction integrated with a p/n GaAs photovoltaic cell.^[^ ^6^, ^146^ ^]^

**Figure 2:**
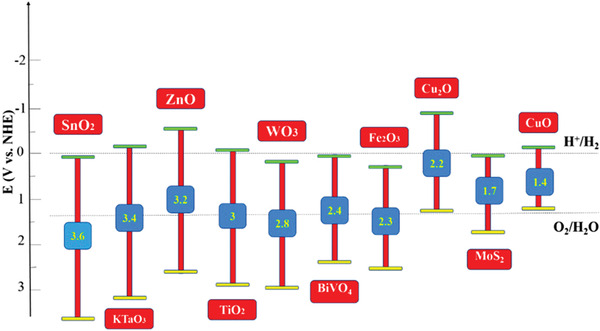
Schematic illustration of the energy bandgaps and band edges of some metal oxide semiconductors at pH = 0.^[^ ^159^ ^]^

Even though these low‐bandgap systems represent promising solar to hydrogen production efficiency, they suffer from low stability in an aqueous solution and are susceptible to corrosion. Thus, surface treatments or protective coatings are necessary for these kinds of materials. Moreover, to reach commercial targets using III–V systems, designing cost‐effective production methods should be regarded as an important issue.^[^ ^147^, ^148^, ^149^ ^]^ Apart from the III–V systems, inexpensive metal oxide semiconductors have been widely investigated for PEC hydrogen production. Low‐bandgap ceramic oxides such as TiO_2_, BiVO_4_, WO_3_, SnO_2_, Fe_2_O_3_, Cu_2_O, and CuO are considered among promising electrode materials for conducting half water‐splitting.^[^ ^141^, ^150^, ^151^, ^152^, ^153^, ^154^, ^155^ ^]^ **Figure** **2** illustrates the band edge position and the bandgap of some promising metal oxides that have attracted great attention during the past decade. These materials have specific conduction band (CB) and valence band (VB) edge potentials with different positions compared to water oxidation–reduction half potentials. For efficient use of metal oxide semiconductors as photoelectrode in PEC cell, either CB edge energy or VB edge position should straddle the water redox potentials or water oxidation potential, respectively.^[^ ^156^, ^157^, ^158^ ^]^ In this regard, bandgap engineering is one of the most effective strategies to improve the electronic and optical properties of nanomaterials for PEC applications such as water splitting.^[^ ^157^, ^159^, ^160^ ^]^ It has been reported that the position of the CB edge depends on the surface charge and the adsorbed dipolar molecules.^[^ ^161^ ^]^ Also, the solution's pH plays a crucial role in determining flat‐band potential (*E*_fb_) and the value of the bandgap.^[^ ^162^ ^]^ It means that the positions of the band edge constantly change by the theoretical voltage of 59 mV per pH unit predicted from the Nernstian behavior. The band structure of semiconductors is further revealed by taking advantage of these features, which can ultimately boost its ability for the split of water.^[^ ^163^, ^164^, ^165^ ^]^

Enhancing charge carrier lifetime and charge mobility are other important issues that should be considered in semiconductor electrodes.^[^ ^166^, ^167^, ^168^ ^]^ Since photogenerated electron–hole are susceptible to recombining with each other, increasing their separation time and mobility are noteworthy issues in this regard. These factors can be investigated via different routes forming heterojunctions by combining different semiconductor materials, using new morphologies including porous structures, and defect introduction into the material structure like dopant agent. There are also some useful approaches for increasing charge mobility in the structure of the semiconductor and surface reactions with water, for example, incorporating conducting materials such as graphene into the structure or increasing the crystallinity of the materials. For promoting charge transfer in the interface between solid and electrolyte, particular coatings or surface treatments can be conducted.^[^ ^14^, ^156^, ^169^ ^]^ As mentioned before, for the photoactive materials, stability, and corrosion resistance in aqueous solution are essential prerequisites. Moreover, the photoelectrode needs to be stable during light absorption and potential fluctuation. These obstacles can be overcome through diverse strategies. As an example, the photoelectrode can be coated with proper materials with high stability in the presence of protons (with or without light). Moreover, modification of semiconductor structure is another approach that should be considered.^[^ ^156^, ^170^, ^171^ ^]^

## 3 General Aspects of CuO

Copper (Cu) is an extremely ductile metal of Group 11 (IB) in the periodic table with atomic number 29. This element, which quickly oxidizes and converts to copper oxide under particular conditions, has two principal oxides depending on the valence state of copper: copper (I) oxide (called cuprous oxide or Cu_2_O) and copper (II) oxide (called cupric oxide or CuO). The unit cell of Cu_2_O contains six atoms, where the oxygen atoms are coordinated by four copper atoms and can crystallize into a cubic structure.^[^ ^172^, ^173^, ^174^, ^175^, ^176^ ^]^ In contrast, CuO has a monoclinic symmetry with the lattice parameters of α = 4.6837 Å, *b* = 3.4226 Å, *c* = 5.1288 Å, β = 99.54°, and α = γ = 90°, where the Cu atom is strongly bonded with four oxygen atoms in an approximately square planar configuration.^[^ ^172^, ^173^, ^177^ ^]^ Despite the very few reports on the use of CuO compared to Cu_2_O for photocatalytic water splitting application, CuO has shown a higher light‐harvesting ability because of its lower bandgap energy than Cu_2_O.^[^ ^178^, ^179^, ^180^, ^181^ ^]^ Also, the theoretical photocurrent densities for CuO and Cu_2_O are 35 and 14.7 mA cm^−2^, respectively, suggesting the superiority of CuO.^[^ ^179^ ^]^ Other properties of copper oxide are given in **Table** **1**.

**Table 1:** Some physical properties of CuO at room temperature (300K).^[^ ^172^, ^187^ ^]^

Density [ρ]	6.31 g cm^−3^
Molar mass	79.55 g mol^−1^
Melting point	1201 °C
Stable phase at 300 K	Monoclinic
Dielectric constant	18.1
Refractive index	1.4
Bandgap (*E*_g_)	1.21–1.7 eV direct
Cu—O bond length	1.96 Å
O—O bond length	2.62 Å
Cu—Cu bond length	2.90 Å
Hole effective mass	0.24 mo
Hole mobility	0.1–10 cm^2^ V^−1^ s^−1^
Specific heat capacity (*C*_p_)	540 J kg^−1^ K^−1^
Thermal conductivity (*k*)	18 W m^−1^ K^−1^
Thermal diffusivity (α × 10^−7^)	51.28 m^2^ s^−1^

Due to the low symmetry of CuO, this material is deemed to have ferroelectric properties.^[^ ^182^ ^]^ Besides, the exchange interaction between Cu^2+^ ions via O^2−^ ions leads to a large but almost constant paramagnetic susceptibility at low temperatures.^[^ ^183^ ^]^ Thus, the giant magnetoresistance, high thermal, and electrical characteristics of CuO have been highlighted, which can be used in a wide range of organic–inorganic nanostructured composites. The CuO nanostructure is one of the most critical inorganic nanostructures that has attracted considerable attention because of its fundamental properties, such as chemical stability, electrochemical activity, and mechanical and optical properties.^[^ ^172^, ^184^, ^185^, ^186^ ^]^

It has been reported that precise control of the morphology with manageable dimensions has a significant effect on the mentioned properties. Moreover, it can be considered as an effective strategy to obtain desirable properties for broadening their potential applications.^[^ ^105^, ^108^ ^]^ Hence, one of the valuable advantages of CuO is the excellent variety of microstructures and the ease of synthesis of this material. Tran et al.^[^ ^173^ ^]^ conducted a systematic study on the important factors that can affect the morphology and size of CuO nanoproducts. Then, they highlighted the effect of various nanostructured forms of CuO on the fundamental properties to pave the way for a deeper understanding of CuO nanomaterials properties.

Cupric oxide is a p‐type semiconductor with a narrow bandgap of around 1.2–1.7 eV. Moreover, the effect of quantum size on CuO nanostructures can change the bandwidth. In several articles, the optical behavior of CuO nanomaterials has been investigated by UV–vis and photoluminescence (PL) techniques.^[^ ^184^, ^185^, ^186^, ^187^, ^188^, ^189^ ^]^

The most common frequent peaks reported for the PL bands of CuO nanostructures appear around 400–600 nm, suggesting that it has expanded from the UV region to the visible light region. As mentioned, CuO is a natural p‐type semiconductor created because of the copper vacancies in the CuO compound. According to recent theoretical calculations, these copper vacancies are the most stable defects in CuO; however, they do not cause any change in the electronic structures of CuO. The reduction of cationic sites in CuO significantly affects the carrier concentration, with hole states placed above the VB. Further information on the optical and electronic properties of CuO nanoparticles is explained in the following sections.

## 4 Photoelectrochemical Properties of Pure CuO

### 4.1 Morphologies

To prepare pristine CuO photoelectrodes with different morphologies, several efforts have been conducted. The CuO can be prepared in the form of nanoparticles,^[^ ^190^ ^]^ nanoleaves,^[^ ^105^ ^]^ nanorods,^[^ ^191^, ^192^, ^193^, ^194^ ^]^ nanosheets,^[^ ^195^ ^]^ nanowires,^[^ ^196^, ^197^, ^198^, ^199^ ^]^ flower‐like,^[^ ^185^ ^]^ and nanofibers.^[^ ^200^ ^]^ Preparing pristine CuO photoelectrodes with high photocurrent response and stability is of great importance because applying this approach obviates the need for preparing hybrid or doped structures. Several issues should be addressed when fabricating photocathodes with just cupric oxide. For instance, synthesis procedures and the operational parameters are responsible on the characteristics of the photoelectrode such as the size of the crystals, surface area, defects, and impurities, which have a significant influence on the final performance of CuO photocathodes. To provide more information around the effect of the type of morphology and fabrication method on photocurrent density, a summary of the photocurrent density values of CuO photocathodes is listed in **Table** **2**. To control the morphology of the nanostructured CuO electrodes, Kushwaha et al.^[^ ^105^ ^]^ designed a facile aqueous‐solution‐based procedure in which two various morphologies of oriented nanosheets and nanoleaves were obtained by changing the concentration of precursor's solution with the photocurrent density of 1.1 and 1.5 mA cm^−2^ at 0 V versus RHE, respectively. The optimum performance of the nanoleaves is a function of several factors such as lower transportation distance for carriers, more reaction sites, and high surface area.^[^ ^105^ ^]^

**Table 2:** Photocurrents of CuO electrodes prepared by different morphology, CuO precursor and synthesis procedures for PEC water splitting

Sn.	Type and morphology of photocathode	Precursor	Fabrication process	Photocurrent density	Ref.
1	CuO nanoleaves	Copper acetate dihydrate	Aqueous solution growth	−1.5 mA cm^−2^ at 0 V versus RHE	^[^ ^105^ ^]^
2	Octahedral flower‐like CuO nanocrystals	Copper (II) sulfate pentahydrate	Coordination‐deposition method	−58.8 µA cm^−2^ at 0.3 V versus RHE	^[^ ^185^ ^]^
3	CuO nanostructured	Cu foil	Chemical bath deposition (CBD)	−1.3 mA cm^−2^ at 0 V versus RHE	^[^ ^201^ ^]^
4	Tree branch‐shaped CuO	Copper(II) nitrate	Hybrid microwave annealing (HMA)	−4.4 mA cm^−2^ at 0 V versus RHE	^[^ ^125^ ^]^
5	CuO nanoparticles	Copper sulfate pentahydrate	Electrodeposition with further annealing	−0.55 mA cm^−2^ at 0.5 V versus RHE	^[^ ^202^ ^]^
6	CuO thin films	CuO target	Radiofrequency (RF) magnetron sputter	−2.5 mA cm^−2^ at 0.5 V versus RHE	^[^ ^96^ ^]^
7	O‐rich CuO nanoparticles	CuO target	RF‐magnetron sputtering	−4 mA cm^−2^ at 0 V versus RHE	^[^ ^124^ ^]^
8	Nanostructured CuO film	Copper (II) acetate	Sol–gel process	−1.5 mA cm^−2^ at 0.5 V versus RHE	^[^ ^203^ ^]^
9	CuO nanostructured	Copper(II) sulfate pentahydrate	Electrochemical deposition	−0.92 mA cm^−2^ at 0 V versus RHE	^[^ ^204^ ^]^
10	CuO 3D nanorods	Cu target	RF sputtering	−3.15 mA cm^−2^ at 0.4 V versus RHE	^[^ ^49^ ^]^
11	CuO nanoparticles	CuO target	RF sputtering + RTP	−1.68 mA cm^−2^ at 0 V versus RHE	^[^ ^99^ ^]^
12	CuO nanoparticles	N/A	Spray‐annealing	‐3.10 mA cm^−2^ at −0.42 V versus RHE	^[^ ^205^ ^]^
13	CuO nanoparticles	Copper (II) chloride dihydrate	Sol–gel dip‐coating process	−0.94 mA cm^−2^ at 0 V versus RHE	^[^ ^75^ ^]^
14	CuO nanofibers	Copper 2‐ethylhexanoate	Electro spinning	−0.16 mA cm^−2^ at 0.4 V versus RHE	^[^ ^200^ ^]^
15	CuO nanowire	CuO target	Thermal oxidation and hydrothermal growth	−0.65 mA cm^−2^ at 0.1 V versus RHE	^[^ ^99^ ^]^
16	CuO nanoparticles	CuO target	RF‐magnetron sputtering	−3.1 mA cm^−2^ at 0 V versus RHE	^[^ ^124^ ^]^
17	CuO nanoparticles	Cu foil	Flame spray pyrolysis	−1.2 mA cm^−2^ at 0 V versus RHE	^[^ ^206^ ^]^
18	CuO porous 2D sheets	Copper nitrate	Electrodeposition + calcination	−3.09 mA cm^−2^ at −0.1 V versus RHE	^[^ ^207^ ^]^
19	CuO nanostructured	Copper sulfate pentahydrate	Potentiostatic deposition + thermal treatment	−0.49 mA cm^−2^ at 0.45 V versus RHE	^[^ ^127^ ^]^
20	CuO nanostructured	Copper(II) sulfate pentahydrate	Electrolysis + annealing	−1.8 mA cm^−2^ at 0 V versus RHE	^[^ ^208^ ^]^
21	CuO nanostructured	Copper sulfate pentahydrate, and copper nitrate	Electrophoresis + annealing at 450 °C	−1.05 mA cm^−2^ at 0.1 V versus RHE	^[^ ^209^ ^]^
22	CuO nanoleaf structure	Copper (II) chloride dihydrate	Aqueous solution under mild refluxing environment	−6.0 mA cm^−2^ at −0.2 V versus RHE	^[^ ^210^ ^]^
23	CuO nanoparticles	Cu target	Reactive DC sputtered + RTP	−6.4 mA cm^−2^ at 0.3 V versus RHE	^[^ ^190^ ^]^
24	CuO nanoparticles	Cu target	Reactive DC sputtering, room temperature	−1.75 mA cm^−2^ at 0.3 V versus RHE	^[^ ^190^ ^]^
25	CuO nanowires	Cu target	Electrochemical two stage growth	−0.35 mA cm^−2^ at 0.05 V versus RHE	^[^ ^211^ ^]^
26	CuO particles	Copper nitrate	Flame spray pyrolysis	−1.2 mA cm^−2^ at 0.5 V versus RHE	^[^ ^64^ ^]^
27	CuO nanoparticles	Copper(II) sulfate	Spinning disk reaction/spin coating	−1.58 mA cm^−2^ at 0.5 V versus RHE	^[^ ^212^ ^]^
28	CuO flower‐like	Copper sulfate pentahydrate	One‐pot microwave synthesis	−0.86 mA cm^−2^ at 0 V versus RHE	^[^ ^213^ ^]^
29	CuO rugby‐like	Copper sulfate pentahydrate	Microwave‐assisted method	−1.15 mA cm^−2^ at 0 V versus RHE	^[^ ^214^ ^]^

Moreover, Li et al.^[^ ^185^ ^]^ synthesized octahedral flower‐like CuO nanocrystals by a coordination‐deposition method and incorporating a modified Fehling reaction that represented an appropriate procedure for large‐scale production. According to the report, changing the concentration of tartrate ions and reaction time has a significant effect on the prepared hierarchical nanostructure. The flower‐like CuO nanocrystals with a porous surface and the bandgap of 1.5 eV exhibited a maximum photocurrent density of 58.8 µA cm^−2^.^[^ ^185^ ^]^

A low‐cost chemical bath deposition (CBD) strategy was introduced by Ray et al.^[^ ^201^ ^]^ to prepare CuO photocathodes. The nanostructured CuO photocathodes were fabricated by immersing Cu foils in an aqueous solution containing 9 mL DI water, 1 mL (NH_4_)_2_S_2_O_8_, and 5 mL NaOH. Next, the mix was placed in a thermostat at 60 °C for 20 h, followed by performing the drying and calcination processes. According to the report, both the calcination and NaOH concentration had a major influence on the performance of CuO photocathodes. The highest photocurrent density (−1.3 mA cm^−2^) was observed for the sample calcined at 200 °C. In this study, the enhancement in photocurrent density was attributed to the improved photogeneration of electron–holes across direct band edges with a most suitable gap of 1.55 eV rather than its inherent indirect‐bandgap nature.^[^ ^201^ ^]^

**Figure 3:**
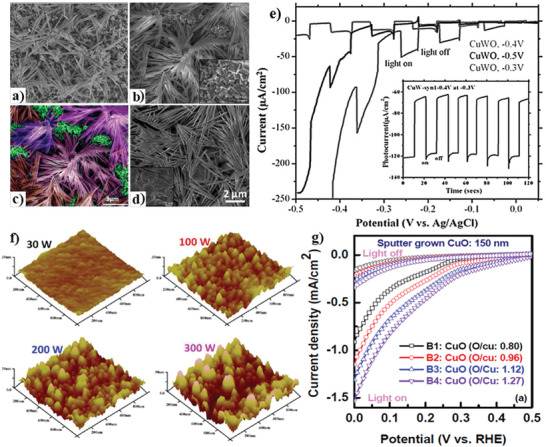
a–d) SEM images of the samples deposited at potentials of −0.3 V (a), −0.4 V (b,c), −0.5 V (d), and e) polarization curves under chopped light for samples deposited at different potentials. The thermodynamic potential for hydrogen evolution should be at −0.855 V and for oxygen and evolution should be at 0.375 V. f) AFM images of samples B1–B4, and g) PEC current–voltage measurements for the B1 sample (deposited at 3 mTorr), the B2 sample (deposited at 8 mTorr), the B3 sample (deposited at 15 mTorr), and the B4 sample (deposited at 40 mTorr). a–e) Reproduced with permission.^[^ ^211^ ^]^ Copyright 2020, Royal Chemical Society. f) Reproduced with permission.^[^ ^96^ ^]^ Copyright 2020, American Chemical Society. g) Reproduced with permission.^[^ ^124^ ^]^ Copyright 2020, American Chemical Society.

Chen et al.^[^ ^211^ ^]^ synthesized copper oxide nanowires via a two‐step electrochemical process without using templates and surfactants. In a recent study, by using a metallic Cu target, the sputtered amorphous copper oxide films were fabricated on the FTO‐coated glass substrates and were electrochemically corroded in 3 m lactic acid and 0.4 m CuSO_4_ electrolytic bath solution. In the next step, CuO nanowires were grown on the corroded samples. The nanowire samples exhibited much better PEC performance compared with the cube‐like samples. The higher surface areas and better contact with the FTO‐coated substrates could be the reasons for the enhanced performance of the CuO nanowires. **Figure** **3**a–d illustrates the SEM images of CuO nanowires deposited at potentials of: a) −0.3 V, b, c) −0.4 V, and d) −0.5 V. By decreasing the potential to −0.5 V, nanowires with the greater size in both radius and length were formed. Moreover, it was found that the increase in the current density by inducing more negative potentials was responsible for raising the growth rate and further effect on the final morphology.^[^ ^211^ ^]^

As can be seen in Figure 3e, the variation of the applied potentials was responsible for the observed changes in nanowires morphology and PEC performance. The highest photocurrent density of −55 µA cm^−2^ was observed for the sample prepared at −0.3 V, while the best performance was recorded for the sample deposited at −0.4 V. The p‐type behavior can be inferred by the presence of cathodic photocurrent and its increase by applying a more negative potential. Cathodic corrosion of CuO led to the existence of high dark currents for all samples. The good stability of the photoelectrode prepared at −0.4 V is represented in the inset image in Figure 3e.^[^ ^211^ ^]^

As mentioned before, several chemical routes have been developed for the synthesis of cupric oxide. Nevertheless, some negative issues regarding the synthesis operation need to be addressed. For example, achieving a highly crystalline CuO structure requires spending several hours for synthesis and further conventional annealing steps that limit the large‐scale production targets. Regarding this issue, Jang et al.^[^ ^125^ ^]^ developed a hybrid microwave annealing (HMA) route for obtaining high‐performance CuO photoelectrodes. In this study, tree‐branch‐shaped CuO photocathodes were prepared by 10 min of microwave irradiation to the deposited Cu–Cu*_x_* O layer placed on the FTO substrate with the presence of silicon as a susceptor. The obtained CuO photocathode represented one of the highest reported photocurrent densities (i.e., −4.4 mA cm^−2^) for the PEC water‐splitting reaction. The reason for the high photocurrent can be the enhanced charge mobility in this unique hierarchical structure, decreased charge recombination rate due to the high purity and crystallinity, and promoted contact with electrolyte due to the presence of high pore volumes and surface area.^[^ ^125^ ^]^

### 4.2 Synthesis Methods

Mahmood et al.^[^ ^202^ ^]^ examined the effect of electrodeposition time on the PEC properties of CuO films and their microstructure. In this study, the CuO films were prepared on ITO substrates by changing the deposition time from 300 to 1800 s and further heat treatment at 550 °C for 2 h. The highest photocurrent density (0.55 mA cm^−2^ at 0.5 V) was reported for the sample deposited at 600 s, suggesting the lowest bandgap (1.4 eV) and low resistance from electrochemical impedance spectroscopy (EIS). The enhancement of the electrodeposition time is responsible for the increased thickness and average grain size. Thus, it has been suggested that this phenomenon promotes absorption due to an increase in the number of CuO particles. This, in turn, results in a higher number of mobile charge carriers and surface roughness. Nevertheless, the larger grains are assumed to have more defects such as oxygen vacancies and porosity, which inhibit the movement of electrons to the surface of the cupric oxides. Therefore, the optimum grain size is required to experience an appropriate photoresponse.^[^ ^202^ ^]^

In another study, Masudy Panah et al.^[^ ^96^ ^]^ investigated the effect of crystalline quality on the photocurrent and photostability of sputtered cupric oxide (CuO) photocathodes. Increasing the magnetron sputtering power enhanced the crystal quality (which is responsible for the enhancement in electron mobility from CuO to the electrolyte), improved separation of photogenerated charge carriers, and increased film surface roughness. As can be seen in Figure 3f, the surface roughness of the film was increased by raising the sputtering power. It is of note that a low nucleation rate and a high growth rate are responsible for the formation of large grain size. By increasing the sputtering power, more particles can reach the substrate surface. So, it results in the enhancement of growth rate at the CuO film and forming a rougher film surface with fewer grain boundaries, which provides a better contact area between the electrode and the electrolyte. In this study, due to high crystallinity and surface roughness, the sample prepared at 300 W sputtering power represented better PEC performance (≈0.92 mA cm^−2^) compared to those prepared at lower sputtering powers. In addition, when the crystallinity of the deposited samples was tuned, the bare 150 nm thin CuO photocathode could retain ≈75% of the initial photocurrent. In this photocathode, increasing the thickness to 500 nm led to further enhancement in photocurrent density (2.5 mA cm^−2^) and photo‐current conversion efficiency.^[^ ^96^ ^]^ In another study, Masudy‐Panah et al. prepared stable and efficient CuO‐photocathodes through oxygen‐rich composition and sputtering of gold–palladium (Au–Pd) nanoparticles on the cupric oxide surface.^[^ ^124^ ^]^ The results showed that changing the composition of copper‐rich CuO to oxygen‐rich CuO has a major effect on the solar to the hydrogen conversion efficiency of CuO‐based photoelectrodes. In this regard, the sputtered O‐rich photocathode exhibited a high photocurrent density of 3.1 mA cm^−2^ at 0 V versus RHE toward the water reduction of, retaining 90% of its initial photocurrent after 20 min. Besides, the O‐rich photoelectrode represents better stability against phase transformation and CuO reduction to Cu_2_O. It is explicated that the formation of an unwanted Cu_2_O phase which significantly influences the photo‐corrosion stability of the photoelectrode can be considerably reduced through in situ materials engineering using O‐rich CuO thin film. Figure 3g shows the PEC current–voltage measurements for the samples deposited with various O/Cu ratio, indicating the influence of CuO compositional change on PEC performance. As can be seen, an increase in the oxygen‐to‐copper ratio improves the photocurrent density because of the enhancement in electron–hole separation and the longer lifetime of photogenerated charge carriers.^[^ ^124^ ^]^

Further deposition of Au–Pd nanoparticles on the surface of O‐rich CuO led to a 25% increase in photocurrent density (4 mA cm^−2^ at 0 V). It was observed that the presence of Au–Pd nanostructures promotes the optical absorption of CuO thin film since these nanoparticles can partially cooperate in the absorption of light and also are prone to scatter the irradiated light that has a positive effect on light harvesting.^[^ ^124^ ^]^

It has been suggested that surface plasmon resonance (SPR) can be responsible for creating a strong electromagnetic field on the noble metal nanomaterials. The light interaction between cupric oxide and nanostructures can promote optical absorptions and charge separation, which subsequently may result in the enhancement of solar conversion efficiency and hydrogen production.^[^ ^124^, ^215^ ^]^

**Figure 4:**
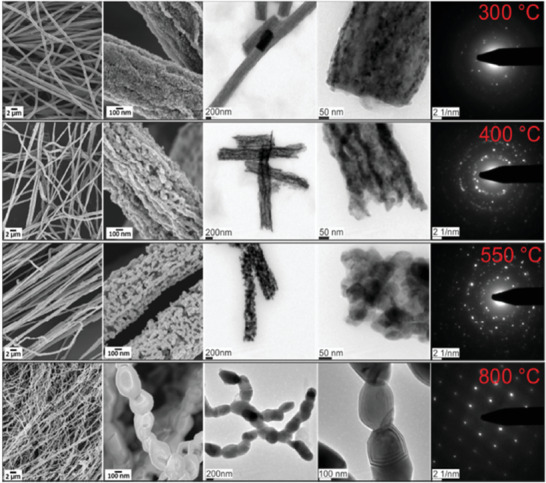
Scanning and transmission electron microscopy in low and high magnification of CuO nanofibers calcined at 300 °C (top row), 400 °C (2nd row), 550 °C (3rd row), and 800 °C (4th row) and the corresponding SAED patterns. Reproduced with permission.^[^ ^200^ ^]^ Copyright 2020, Wiley‐VCH.

Hosseini et al.^[^ ^214^ ^]^ investigated the effect of the duration of microwave (MW) irradiation on photocatalytic water splitting activity of CuO thin film. They prepared four samples at various irradiation durations (15, 30, 60, and 90 min), which were named CuO‐X, where X stands for irradiation time. They reported the highest achieved photocurrent density of synthesized samples (−1.15 mA cm^−2^ at 0 V versus RHE) belongs to the CuO‐60 sample with a hierarchical rugby‐ball‐like CuO structure. Higher specific surface area because of the optimized duration of microwave irradiation for this sample, the crystalline structure, unique hierarchical morphology, and uniform distribution of nanocrystalline particles alongside the CuO nanosheets were the reasons for superior PEC performance of CuO‐60 photocathode. Such unique morphologies can provide an excellent pathway for a more efficient transfer and/or separation process. Recently, Einert et al.^[^ ^200^ ^]^ prepared fibrous CuO photocathodes via the electrospinning method and calcination treatment. **Figure** **4** represents the SEM and TEM images of the electro‐spun CuO nanofibers prepared by different calcination temperatures. According to this study, by increasing the annealing temperature, crystalline domains can develop because of the improved conductivity. Besides, increasing the calcination temperature decreases the number of defect sites that significantly affects the recombination of photoexcited electron–holes. The reason is that a lower number of trap states result in better separation of charge carriers. Therefore, the sample prepared at 300 °C, compared to the CuO fibers calcined at 800 °C, exhibited 5‐times increased photocurrent density of −14.9 µA cm^−2^ at 0.375 V versus RHE. It has been claimed that increasing the film thickness was responsible for achieving photocurrents up to −0.16 mA cm^−2^ at 0.4 V versus RHE.^[^ ^200^ ^]^ Lim et al.^[^ ^179^ ^]^ also successfully prepared the CuO electrodes with a thickness of 300 nm through the sol–gel spin coating method, which exhibited a photocurrent density of −0.35 mA cm^−2^ for CuO at 0.05 V versus RHE, while the photocurrent density value of CuO electrode was decreased with increasing film thickness to 600 nm (−0.33 mA cm^−2^). Thus, the results showed that CuO photoelectrodes were more stable than Cu_2_O in PEC cells.

## 5 Photoelectrochemical Properties of CuO Composites

### 5.1 Incorporating Metallic Elements into the CuO Structure

Incorporating metallic elements into the CuO structure has a major influence on the morphology and stability of photoelectrodes in the electrolyte. For instance, Tang et al.^[^ ^216^ ^]^ showed that alloying CuO with titanium (Ti) promotes stability in an aqueous solution due to the formation of Ti–O bonds on the surface. It has been noted that incorporating Ti into CuO negatively affects the photocurrent density (80% reduction compared to that of pure CuO).^[^ ^216^ ^]^

Guo et al.^[^ ^54^ ^]^ fabricated CuO/Pd photocathode with a bandgap energy of 1.56 eV and high PEC performance toward the hydrogen evolution reaction (HER). The electrodes were prepared by chemical to synthesize Cu nanoparticles, followed by the spin coating process on the FTO substrate. Afterward, 10‐layered CuO films were obtained by thermal treatment in an air flowing furnace at 550 °C for 4 h. The CuO pristine photocathode exhibited an approximate 19% decrease in photoactivity (after reaction for 10 h under light radiation) due to the formation of Cu_2_O on the part of the surface. By further photoassisted electrodeposition of Pd nanoparticles, CuO/Pd photocathodes were formed with the highly enhanced photocatalytic stability of CuO.^[^ ^54^ ^]^ The study of current–potential curves of pristine CuO and CuO/Pd photocathodes showed more reduction current during the illumination at 0.1 V for 15 min in 0.1 m KCl containing 0.5 mm Na_2_PdCl_4_, which is due to the low HER overpotential of the palladium co‐catalyst. In this study, the samples were subjected to EIS to investigate electron transfer activity across the CuO/electrolyte interface. In the Nyquist plots, the semicircle observed in the high‐frequency region corresponds to the CuO/FTO and CuO/FTO contact resistance. Since the contact resistance and the capacitance of CuO/FTO are independent of illumination and palladium addition, this semicircle remained almost unchanged.

The interface, which can be shown by CuO/FTO interfacial capacitance impedance of the CuO/solution interface, can be investigated via the low‐frequency semicircle, indicating the charge transfer resistance (*R*_ct_) across the CuO/solution interface by its diameter. According to the CuO/Pd plot, *R*_ct_ under the dark condition is much larger than in the illumination, which is due to the presence of photogenerated electrons across the CuO/electrolyte interface. Another evidence for the positive effect of Pd modification in charge transfer across the electrolyte is the value of *R*_ct_ (108 U cm^2^) on the CuO/Pd electrode, which is much lower than that of the CuO electrode (258 U cm^2^).^[^ ^54^ ^]^

### 5.2 Heterojunction with Oxide Semiconductors

The PEC performance of CuO electrodes can be improved with the formation of metal oxide heterostructures such as CuO/TiO_2_,^[^ ^217^ ^]^ CuO/ZnO,^[^ ^65^ ^]^ CuO/Cu_2_O,^[^ ^218^ ^]^ and CuO/Al_2_O_3_.^[^ ^219^ ^]^ One of the promising heterojunctions to consider is CuO/Cu_2_O photocathodes, which have attracted much attention because of enhanced charge transportation and photostability, as well as simple fabrication via several approaches.

Zhang et al.^[^ ^220^ ^]^ prepared highly stable CuO composite photoelectrodes via a simple two‐step electrochemical technique consisting of electrodeposition of a copper film on an ITO glass substrate perused by anodizing step and further annealing to achieve Cu_2_O/CuO composite. The final composite was composed of a protective thin film of CuO on the thin layer of Cu_2_O. According to the report, the preferred orientation of chemical composition and crystalline degree of the materials has a major influence on the PEC H_2_ production. Regarding this issue, the Cu_2_O sample with (220) orientation represented the best PEC performance (photocurrent density of 1.54 mA cm^−2^) among all fabricated composites. By comparing the stability of the prepared photocathode with that of the pristine Cu_2_O electrode (30.1%), the composite electrode represented enhanced stability of 74.4%. In this structure, the top layer of CuO acted as both protective layers against photo‐corrosion and charge carrier recombination inhibitor.^[^ ^220^ ^]^

**Figure 5:**
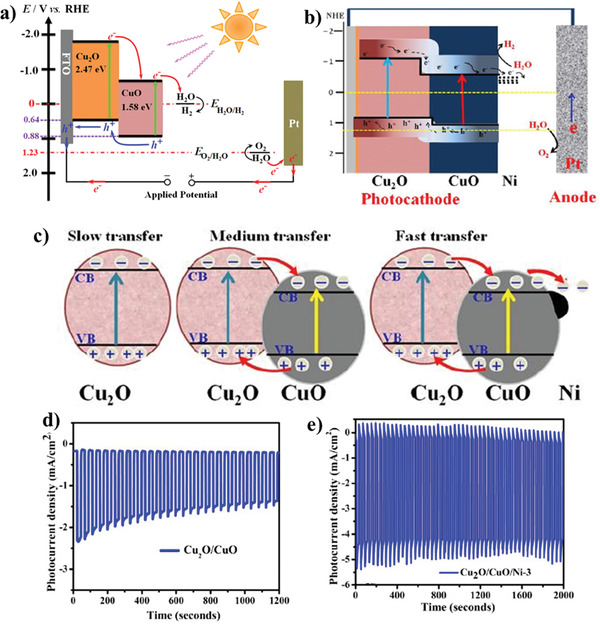
a) The schematic representation of the energy band diagram of the Cu_2_O/CuO bilayer composite in contact with a solution during PEC HER. b) Proposed mechanism of PEC water splitting. c) The energy band structure of Cu_2_O, Cu_2_O/CuO, and Cu_2_O/CuO/Ni. d,e) Stability measurement of Cu_2_O/CuO and Cu_2_O/CuO/Ni‐3 (up to 3 deposition cycles), respectively. a) Reproduced under the terms of the CC‐BY Creative Commons Attribution 4.0 International license (https://creativecommons.org/licences/by/4-0).^[^ ^35^ ^]^ Copyright 2020, The Authors, published by Springer Nature. b–e) Reproduced with permission.^[^ ^208^ ^]^ Copyright 2020, Royal Society of Chemistry.

In another study, Yang et al.^[^ ^35^ ^]^ made an effort to fabricate a Cu_2_O/CuO photocathode. They prepared Cu_2_O/CuO bilayer composite via electrodeposition method and further thermal oxidation, which lead to the photocurrent density of 3.15 mA cm^−2^ at 0.40 V versus RHE in 1.0 m KOH solution. Schematic illustration of FTO coated Cu_2_O/CuO heterojunction under visible light irradiation is represented in **Figure** **5**a. CB edges of both CuO and Cu_2_O are located at a more negative potential domain, which is fundamental for reducing water molecules. Therefore, the photoinduced electrons of Cu_2_O can be transferred to the CB of CuO, which promotes charge separation and PEC water splitting ability. Regarding the synthesis procedure, preparation of the narrow‐bandgap CuO layers on Cu_2_O substrate enhances the amount of solar light absorption. Moreover, this bilayer hybrid structure has exhibited better charge carrier density and transportation.^[^ ^35^ ^]^ One of the promising hybrid structures for PEC water splitting is ZnO/CuO heterojunction, which can be prepared in diverse morphologies. One approach in this regard is to prepare a 1D CuO structure and further the addition of ZnO to the surface. The 1D/CuO nanostructures with the enhanced surface‐to‐volume ratio and light‐harvesting performance can have a positive influence on PEC water splitting.

Kargar et al.^[^ ^206^ ^]^ prepared ZnO/CuO heterojunction branched nanowires (b‐NWs) via a facile and cost‐effective procedure. Using thermal oxidation growth method, the CuO nanowire arrays were grown on copper substrates, followed by depositing thin ZnO seeding layer on cupric oxide NWs by RF magnetron sputtering pursued by a hydrothermal process for the formation of ZnO nanowires. The energy band diagram of ZnO/CuO heterojunction branched nanowires in contact with the solution showed at a reversed biasing potential of −0.45 V. At this potential, downward movement of the ZnO energy levels caused more band bending at the CuO–ZnO junction, which is accompanied by the enhanced charge separation and mobility. Besides, the other reason for enhanced photocurrent was the decreased barrier at the ZnO–electrolyte junction. In this study, the branched NWs with longer and denser CuO NW cores represented better photocathodic current. The fabricated branched NWs showed higher photocathodic current and better photoresponse compared to the ZnO‐coated CuO (core/shell) NWs, due to the increased surface area and improved gas evolution.^[^ ^206^ ^]^ In another study, Shaislamov et al.^[^ ^65^ ^]^ employed a simple strategy for fabricating highly stable hierarchical p‐CuO/ZnO nanorod. In this study, first, the direct growth of Cu nanorods (NRs) was applied by electrodeposition method using an ion track‐etched polycarbonate filter as a template. Next, the CuO NRs were obtained by a low‐temperature annealing process in ambient air, followed by deposition of ZnO seed layer via dip‐coating several times. Using the hydrothermal method, uniform growth of ZnO NR branches occurred on the sidewalls of each CuO trunk NR. The presence of a dense and uniformly grown ZnO NR layer between the CuO/electrolyte interfaces is responsible for reaching stability of up to 90%. The high stability was achieved because of the appropriate protection of the CuO NR trunk from direct contact with electrolyte. However, in the case of photocurrent efficiency, the pristine CuO NR electrode exhibited better performance than CuO/ZnO NR photoelectrodes.^[^ ^65^ ^]^

An alternative approach for preparing CuO/ZnO heterostructure has been conducted by Zhao et al.^[^ ^47^ ^]^ In this research, CuO/ZnO core/shell NW arrays were designed via the facile thermal oxidation of copper foil for preparing cores. Next, the saturated ethanol solution containing zinc acetate was covered on the CuO NWs. Afterward, it was subjected to further thermal treatment at 350 °C for 20 min in the air. The obtained photoelectrode demonstrated a 0.71% photon‐to‐hydrogen conversion efficiency for PEC water splitting.^[^ ^47^ ^]^

According to the report by Ng et al.,^[^ ^221^ ^]^ PEC performance of the WO_3_/CuO heterojunction photoelectrodes was on par with the pristine CuO electrodes under light illumination. Both CuO and composite electrodes represented PEC activity at a negative bias potential.

Regarding that, the FTO/CuO/WO_3_ photoelectrode shows higher stability, in this study, the pristine CuO photocathode represented better performance in the reduction of hydrogen ions as opposed to heterojunction structure. This result may be related to the CB edge position of WO_3_ located at a more positive region (−0.26 V versus NHE at pH 7) concerning the reduction potential of water that is −0.41 V versus NHE at pH 7.^[^ ^221^ ^]^

Dubale et al.^[^ ^208^ ^]^ demonstrated the highly efficient PEC performance of Cu_2_O/CuO heterojunction decorated with nickel co‐catalyst. The obtained photocathode was fabricated via the electrolysis deposition, thermal annealing in air, and spin‐coating technique. Figure 5b,c illustrates the proposed water reduction mechanism at prepared photocathode and the energy band structure of pristine Cu_2_O, Cu_2_O/CuO heterojunction, and Cu_2_O/CuO/Ni photocathode.

Compared to pristine CuO, the better photocurrent density and photostability of the Cu_2_O/CuO heterojunction is attributed to the synergistic effect and improved crystallinity. As mentioned before, in Cu_2_O/CuO hybrid structure, the light‐harvesting efficiency is enhanced. Besides, the electron–hole recombination rate decreases and leads to an enhancement in PEC water splitting activity. Decorating the heterostructure of Cu_2_O/CuO with nickel led to achieving a high photocurrent density of −4.3 mA cm^−2^ compared to that of pristine Cu_2_O/CuO (−2.1 mA cm^−2^). The addition, the presence of nickel on the surface of Cu_2_O/CuO led to the fast transfer of photogenerated electrons into the aqueous solution, as well as surface stabilization. It was observed that nickel with no bandgap energy and high electrocatalytic activity provides a very low HER overpotential. Furthermore, electron injection from CuO into the Ni cocatalyst is responsible for shifting the Ni Fermi level to a more negative potential near the CB level of CuO.^[^ ^208^ ^]^ Figure 5d,e represents the stability measurement of Cu_2_O/CuO and Cu_2_O/CuO/Ni‐3 (up to three deposition cycles), respectively. The pristine Cu_2_O/CuO photoelectrode represented a gradual decrease in photocurrent density (from −2.1 mA cm^−2^) for the first few seconds under chopped illumination at 0 V versus RHE for 20 min. Meanwhile, modifying the Cu_2_O/CuO photocathode surface with a nickel layer led to considerable improvement in photostability. According to a study conducted by Oh et al.,^[^ ^58^ ^]^ enhanced photostability of the CuO photoelectrode was observed by doping CuO seed layers with nickel via the modified chemical bath deposition (M‐CBD) process and spin coating technique. In this work, doping the seed layers with nickel and further growth of CuO led to the diffusion of nickel into the CuO crystal structure. This phenomenon decreased the dark current by preventing the reduction of the cupric oxide to copper.

**Figure 6:**
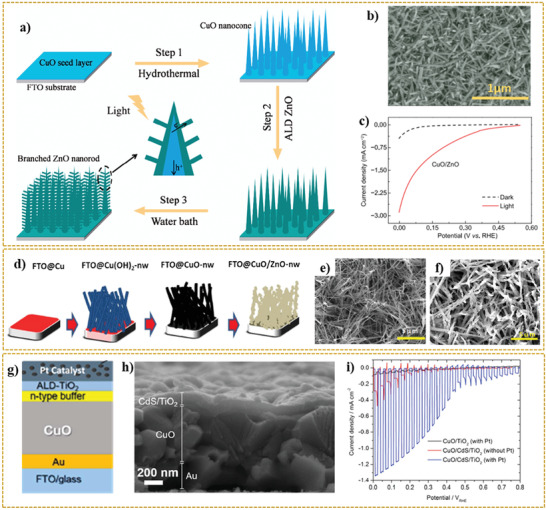
a) The growth process of 3D CuO nanocone/ZnO nanorod arrays. b) SEM images of CuO/ZnO junctions at water bath of 75 min. c) LSV curves of CuO/ZnO (37 cycles, 55 min) junctions in both of dark and light conditions. d) Schematic representation of CuO/ZnO NW heterostructure film synthetic process. e,f) SEM images of CuO‐NW (e) and CuO/ZnO‐NW (f). g) Structure of the CuO/CdS/TiO_2_ photocathode. h) Cross‐sectional SEM images of the CuO/CdS/TiO_2_ photocathode. i) Current density–potential curves of CuO/TiO2 (with Pt) and CuO/CdS/TiO2 with and without Pt‐catalyst measured in 1 M phosphate buffer (pH = 7) under chopped illumination from simulated sun illumination. a–c) Reproduced with permission.^[^ ^222^ ^]^ Copyright 2020, Science China Press/Springer Nature. d–f) Reproduced with permission.^[^ ^223^ ^]^ Copyright 2020, Springer Nature. g–i) Reproduced with permission.^[^ ^224^ ^]^ Copyright 2020, American Chemical Society.

With the improved crystallinity, photogenerated electrons as minority carriers effectively induced the photostability by rapid transfer to the electrode surface.^[^ ^58^ ^]^ Wu et al.^[^ ^222^ ^]^ synthesized 3D p‐CuO/n‐ZnO heterojunction nanoarrays that were incorporated as a photocathode in PEC cells. **Figure** **6**a shows the synthesis procedure employed for this purpose. This procedure consists of a hydrothermal process in advance for preparing CuO nanocone arrays, the atomic layer deposition (ALD) method for depositing ZnO seed layers, and further growth of ZnO nanorod branches via a water bath reaction process. Figure 6b displays the SEM nanostructures of CuO/ZnO nanocomposite in 75 min water bath time. In this study, the ratio of the photocurrent to dark current density for CuO nanocones was 2.7. Meanwhile, for the CuO/ZnO nanostructure, a higher number of 6.4 is reported that corresponds to improved PEC performance due to the effective electron–hole separation, enhanced interface charge mobility, and increased carrier lifetime in this heterojunction system.^[^ ^222^ ^]^ The linear sweep voltammetry (LSV) curves of CuO/ZnO (37 cycles, 55 min) heterojunction photocathodes in both dark and light condition are shown in Figure 6c, representing the current density of 0.9 mA cm^−2^ at 0.2 V versus RHE. In this respect, the existence of photocathodic current peak is attributed to the consequent recombination of photogenerated electron–holes and their accumulation at the interface of electrode/solution. When the light is switched off, a relatively weak dark current peak appears. Thus, by reaching a steady‐state situation of charge carrier recombination, the *J*–*V* curve stabilized accordingly.^[^ ^222^ ^]^

Tsege et al.^[^ ^223^ ^]^ fabricated CuO/ZnO nanowire heterojunction with an outstanding photocurrent density of 8.1 mA cm^−2^ at 0V versus RHE, which showed 13.6% enhancement, compared to that of pristine CuO NW. The schematic illustration of the synthetic procedure of the CuO/ZnO NW heterostructure film can be observed in Figure 6d.

In this research, the fabrication process began with the electrodeposition of Cu film on FTO glass followed by subsequent immersion in a solution containing 0.12 m (NH_4_)_2_S_2_O_8_ and KOH (25:1 molar ratio), which resulted into the formation of Cu(OH)_2_ NWs. Then, the CuO film was prepared via the thermal annealing of Cu(OH)_2_ NW at 450 °C in the air for 1 h. To fabricate CuO/ZnO‐NW heterostructure photoelectrodes with three coating layers, the CuO film was dip‐coated with a solution having well‐dispersed zinc oxide nanoparticles with further annealing process at 450 °C for 1 h. The Cu(OH)_2_ NWs oriented with a diameter of 300 nm randomly and approximate length between 2.5 and 5.5 µm have a uniform distribution on an FTO glass substrate. Figure 6e shows the curly shape morphology of prepared CuO NWs with approximately similar size for length and diameter. The presence of zinc oxide nanoparticles on the surface of CuO NW arrays can be seen in the SEM image in Figure 6f.

Titanium dioxide (TiO_2_) is another promising material to couple with CuO, enabling the formation of heterostructure material. Forcade et al.^[^ ^225^ ^]^ prepared CuO/TiO_2_ nanocomposite by combining nanocrystalline TiO_2_ and CuO powders followed by a deposition step via the “doctor blade” technique. According to this study, the photoexcited electrons in CuO can occupy a lower energy state in TiO_2_. It has been mentioned that enhanced electron collection can be caused by decreasing the size of CuO nanocrystals since this approach reduces the recombination rate of the charge carriers. Furthermore, decreasing the crystallite size can rise CuO CB concerning that of TiO_2_ due to the quantum effect.^[^ ^225^ ^]^

Septina et al.^[^ ^224^ ^]^ refuted the previous claims about the source of generated photocurrent from pristine unprotected CuO films. Although many researchers believe that hydrogen production reactions are responsible for the production of photocurrents, Septina et al.^[^ ^224^ ^]^ proposed that the observed current is because of the photo‐corrosion of cupric oxide in the copper. In other words, the photogenerated electrons are responsible for the reduction of CuO rather than participating in the hydrogen evolution reaction. In this study, pristine CuO thin films were prepared via the facile electrodeposition technique and further annealing steps. Because of observing a photocurrent density up to 1.16 mA cm^−2^ (at 0.4 *V*_RHE_), the hydrogen faradaic efficiency was measured around 0.01%, suggesting the main source of the observed photocurrent density is photo‐corrosion. Accordingly, as shown in Figure 6g,h, a protective layer consisting of TiO_2_ was placed in contact with the electrolyte to inhibit the corrosion of CuO thin film. To promote the performance, a CdS buffer layer between the CuO and TiO_2_ formed the heterojunction. The system containing buried junctions was further developed by platinum as a hydrogen evolution catalyst via the photo‐electrodeposition method. As shown in Figure 6i, the final obtained system represented high stability against the photo‐corrosion with faradaic efficiencies close to 100%.^[^ ^224^ ^]^

Ha et al.^[^ ^226^ ^]^ investigated a CuO nanorod/Al_2_O_3_ heterostructure photoelectrode and its performance in PEC H_2_ production. The hybrid structure is formed by thermal evaporation and further deposition of an aluminum layer on the CuO NRs, followed by an oxidizing step in the air to fabricate a 2 to 10 nm Al_2_O_3_ layer on the substrate. In this study, the photocurrent density of −2.26 mA cm^−2^ (−0.55 V versus SCE) is associated with the Al_2_O_3_ layer, which accepts electrons from CuO in this heterojunction system.^[^ ^226^ ^]^

In this study, the fabrication method and PEC water splitting performance of diverse CuO‐based photocathodes were investigated. **Table** **3** represents the photocurrents of CuO electrodes prepared by different synthesis procedures for PEC water splitting. The contents of this table are divided into the doped CuO and CuO composites photoelectrodes. Thus, from Table 3, it can be concluded that the morphology of the synthesized electrodes plays a vital role in the CuO PEC properties. Moreover, introducing elements into the CuO structure and the formation of heterojunction can be regarded as efficient approaches to increase the output current density and the amount of hydrogen production. For instance, decorating the CuO photoelectrodes with noble metals such as Pt and Au can improve the visible light absorption ability. Therefore, the mentioned factors lead to the promotion of the charge transfer and preventing the electron–hole recombination. Although some of the reported current densities are low however, it seems encouraging and paves the way for future advances in this category of materials. Likewise, the correlation between the nature of the material and the PEC properties of CuO‐based photoelectrodes can be considered to give some useful information on factors that affect the PEC capability.

**Table 3:** Photocurrents of doped CuO, and CuO composites photoelectrodes prepared by different synthesis procedures for PEC water splitting

Sn.	Type and morphology of photocathode	Fabrication process	Photocurrent density	Ref.
1	2 at% Li doped CuO	Flame spray pyrolysis	1.69 mA cm^−2^ at −0.55V versus Ag/AgCl	^[^ ^227^ ^]^
2	Ni/CuO fibers	Electrospinning	2.6 mA cm^−2^ at −0.5V versus Ag/AgCl	^[^ ^228^ ^]^
3	Al‐incorporated CuO (CuO:Al)	Radio frequency	3.7 mA cm^−2^ at 0V versus RHE	^[^ ^229^ ^]^
4	P(CuO/CuO:Al)/nZnO:Al/TiO_2_/Au–Pd	Radio frequency	5.4 mA cm^−2^ at 0V versus RHE	^[^ ^229^ ^]^
5	Cu_2_O/CuO nanorods	Physical vapor deposition	0.24 mA cm^−2^ at −0.5 V versus Ag/AgCl	^[^ ^230^ ^]^
6	Ni‐doped CuO nanorods	Chemical bath deposition	1.75 mA cm^−2^ at −0.55V versus SCE	^[^ ^231^ ^]^
7	Cu/Cu_2_O/CuO nanowires	Thermal oxidation	1.8 mA cm^−2^ at 0V versus RHE	^[^ ^118^ ^]^
8	CuO/Cu_2_O	Electrodeposition + annealing	0.451 mA cm^−2^ at −0.3V versus Ag/AgCl	^[^ ^232^ ^]^
9	CuO/Cu_2_O grass appendage‐like	Electrodeposition	1.44 mA cm^−2^ at −0.7 V versus Ag/AgCl	^[^ ^233^ ^]^
10	CuO/SrTiO_3_ nanostructure	Sol–gel spin‐coating	1.85 mA cm^−2^ at −0.9 V versus SCE	^[^ ^234^ ^]^
11	Cu_2_O/CuO/WO_3_	Electrodeposition and annealing	1.9 mA cm^−2^ at 0 V versus RHE	^[^ ^235^ ^]^
12	CuO/CuWO_4_	Electrodeposition and annealing	2.8 mA cm^−2^ at 0 V versus RHE	^[^ ^235^ ^]^
13	Cu_2_O/CuO composite	Electrodeposition followed by anodization	1.54 mA cm^−2^ at 0 V versus RHE	^[^ ^220^ ^]^
14	Cu_2_O/CuO bilayered composites	Electrodeposition and a subsequent thermal oxidation	3.15 mA cm^−2^ at 0.4 V versus RHE	^[^ ^35^ ^]^
15	ZnO/CuO branched nanowires	Thermal oxidation and hydrothermal growth methods	1.3 mA cm^−2^ at 0 V versus RHE	^[^ ^206^ ^]^
16	CuO/ZnO nanorod nano branch	Direct thermal oxidation of Cu nanorods	0.9 mA cm^−2^ at 0.5 V versus RHE	^[^ ^65^ ^]^
17	CuO/ZnO core/shell heterostructure NWs	Oxidation method followed by thermal decomposition	1.54 mA cm^−2^ at 1 V versus RHE	^[^ ^47^ ^]^
18	p‐CuO/n‐ZnO heterojunction nanoarrays	Water bath reaction process together with the atomic layer deposition (ALD) technology	0.9 mA cm^−2^ at 0.2 V versus RHE	^[^ ^222^ ^]^
19	CuO/ZnO nanowire	Electro‐deposition of Cu Film followed by a subsequent chemical oxidation and dip‐coating methods	8.1 mA cm^−2^ at 0 V versus RHE	^[^ ^223^ ^]^
20	CuO/CdS thin film	Chemical bath deposition followed by ALD‐TiO2 onto the CuO thin film	1.68 mA cm^−2^ at 0 V versus RHE	^[^ ^224^ ^]^
21	CuO nanorod/Al_2_O_3_	Modified‐chemical bath deposition followed by thermal evaporation	2.26 mA cm^−2^ at 0.55 V versus, SCE	^[^ ^226^ ^]^
22	CuO nanofibers	Electrospinning	0.16 mA cm^−2^ at 0.4 V versus RHE	^[^ ^200^ ^]^
23	Ti‐alloyed CuO	RF magnetron co‐sputtering	0.2 mA cm^−2^ at −0.3 V versus RHE	^[^ ^216^ ^]^
24	CuO/Pd nanoparticles	Solution synthesis, spin‐coating, and thermal treatment processes	0.8 mA cm^−2^ at 0.44 V versus RHE	^[^ ^54^ ^]^
25	WO_3_/CuO heterojunction	Electrodeposition	0.18 mA cm^−2^ at −0.7 V versus RHE	^[^ ^221^ ^]^
26	Cu_2_O/CuO decorated with nickel	Electrolysis deposition, thermal annealing in air and spin‐coating processes	4.3 mA cm^−2^ at 0 V versus RHE	^[^ ^208^ ^]^
27	CuO photoelectrode with Ni‐doped seed layer	M‐CBD process	1.33 mA cm^−2^ at 0 V versus RHE	^[^ ^58^ ^]^
28	CuO/Cu_2_O shell/core heterostructure	Electrochemical anodization + annealing	1.9 mA cm^−2^ at 0.3 V versus Ag/AgCl	^[^ ^218^ ^]^
29	Cu_2_O/CuO	Electrolysis+ annealing	2.1 mA cm^−2^ at 0 V versus RHE	^[^ ^208^ ^]^
30	Ni decorated Cu_2_O/CuO	Electrolysis + hydrothermal + spin‐coating	4.3 mA cm^−2^ at 0 V versus RHE	^[^ ^208^ ^]^
31	TiO_2_ in Cu_2_O–CuO heterojunction	Anodising Cu foil: TiO_2_/CuO	2.4 mA cm^−2^ at 0 V versus RHE	^[^ ^236^ ^]^
32	Cu_2_O/CuO nanowires	Calcination of the anodized Cu_2_O	1.3 mA cm^−2^ at 0 V versus RHE	^[^ ^236^ ^]^

### 5.3 Heterojunction with 2D Materials

#### 5.3.1 CuO/2D Carbon Material Heterojunctions

In recent years, 2D carbon material has been extensively used in composite materials to improve the electrical and optical properties of its host matrix.^[^ ^237^, ^238^, ^239^, ^240^, ^241^, ^242^, ^243^, ^244^, ^245^ ^]^ The inherent properties of these 2D materials, including high specific surface area and excellent electron‐transport properties, makes them a unique option for photocatalyst applications, especially PEC water splitting. These materials can form a new class of multiphase materials with high performance.^[^ ^246^, ^247^, ^248^, ^249^, ^250^, ^251^, ^252^ ^]^ Moreover, in light of simple and various fabrication process of cupric oxide as well as various morphologies, it can be easily composited with 2D carbon material. Thus far, different methods have been suggested for the synthesis of this composite.^[^ ^253^, ^254^, ^255^, ^256^, ^257^, ^258^, ^259^, ^260^, ^261^, ^262^ ^]^ Among various techniques for merging the compounds between these two materials, we can name the integration of CuO–reduced graphene oxide (rGO). The process was conducted by the dissolution of CuSO_4_·5H_2_O in deionized water and GO solution through the co‐precipitation method,^[^ ^263^ ^]^ embedding CuO islands in the graphene film, where graphene was grown on polycrystalline Cu foil in a furnace by CVD procedure,^[^ ^264^ ^]^ and synthesis of leaf‐like CuO on graphene sheets by a hydrothermal method.^[^ ^265^ ^]^

Xiong et al.^[^ ^266^ ^]^ also reported the successful fabrication of CuO/GO/CuO sandwich‐like nanosheets using a combined process of assembly, reduction, and consolidation. In this study, GO aqueous dispersion with a concentration of 1 mg mL^−1^ was mixed with sodium dodecyl sulfate solution (0.5 m) and urea. Then, the Cu(NO_3_)_2_·3H_2_O solution was added dropwise under vigorous stirring to the mixture. Finally, the resulting mixture was stirred in a sealed glass flask at 90 °C for 16 h, and CuO/GO/CuO nanosheet solution was obtained once the product cooled to room temperature.^[^ ^266^ ^]^

According to several reports, the development of heterojunction between graphene and copper oxide can effectively suppress the recombination of charge carriers and accelerate the electron transfer, leading to a significant enhancement of the photocatalytic properties. One of the essential agents to improve the aforementioned features is the graphene electron acceptor in the heterostructure.^[^ ^267^ ^]^ In addition to the water splitting applications of CuO/2D graphene composites, they have been used in various applications including degradation of methylene blue and H_2_ evolution from water due to the excellent photocatalytic properties.^[^ ^268^, ^269^, ^270^, ^271^, ^272^, ^273^, ^274^ ^]^ For example, Jingqi et al.^[^ ^275^ ^]^ developed a composite of CuO–Cu_2_O–Cu nanorod‐decorated reduced graphene oxide (CuNRs‐rGO) using one‐pot green hydrothermal heating for applications in photocurrent generation. In this study, GO was prepared through a modified Hummer's method. To fabricate CuNRs‐rGO composite, under alkaline conditions, 8 m NaOH solution was added dropwise to the GO and copper acetate (Cu(OAC)_2_) solution. Finally, the mixed solution was kept in the autoclave for 3 h at 180 °C.^[^ ^275^ ^]^ According to the absorption spectra reported by this research group, with the formation of the CuNRs‐rGO composite, the π–π* and n–π* transition bands disappeared. These bands correspond to the aromatic C=C and C=O bands at 230 and 300 nm in GO, respectively. In contrast, a broad peak at 400 nm appeared, indicating a high optical absorption of the CuNRs‐rGO composite relative to the GO. Moreover, it was found that the presence or absence of GO can have a significant effect on the morphology of copper and its oxides. The absence of graphene oxide resulted in the formation of CuO irregular micron size particles (CuMPs). In comparison, in the presence of GO, the nanorod morphology (CuNRs‐rGO) was formed. Investigations on the photocurrent response of the rGO, CuMPs, and CuNRs‐rGO samples revealed the current densities of about 0.04, 0.11, and 0.23 µA cm^−2^ were obtained, respectively, at 0 V versus Ag/AgCl reference electrode in 1 m Na_2_SO_4_ electrolyte under the white light irradiation (100 mW cm^−2^). This was due to the ability of higher light absorption and the formation of a heterojunction structure in the CuNRs‐rGO sample than the counterparts.^[^ ^275^ ^]^

In addition, a sudden cathodic sharp photocurrent spikes in the chopped‐light voltammogram under illumination due to the rGO's excellent charge generation ability within the composite. Moreover, because of the high recombination rate of charge carrier in the composite, the photocurrent decreases immediately, where the cathode current again offsets this reduction. Overall, this rise and fall indicate a better PEC performance of CuNRs‐rGO composite than CuMPs and pure rGO samples. This improvement denotes the decisive role of graphene in the composite, as it plays a distinct electron acceptance role and facilitates the photogenerated charge transfer in the CuO/rGO nanostructure.^[^ ^275^ ^]^

**Figure 7:**
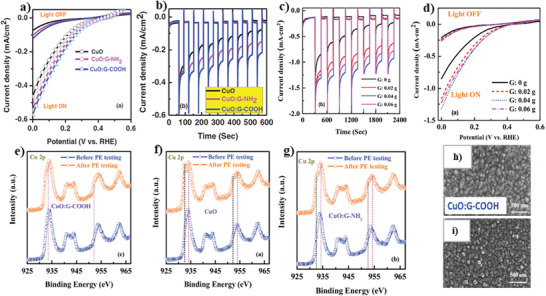
a) LSV of CuO, CuO:G‐NH2, and CuO:G‐COOH photocathodes. b) Stability of CuO, CuO:G‐NH2, and CuO:G‐COOH photocathodes. c) Linear‐sweep voltammograms of CuO:G‐COOH photocathode with different amounts of graphene. d) Stability of CuO:G‐COOH photocathode with different amounts of graphene. e–g) XPS spectra of Cu 2p core level spectra from synthesized photocathode. h,i) Surface morphology of CuO:G‐COOH thin films before and after photocurrent measurement. a–i) Reproduced under the terms of the CC‐BY Creative Commons Attribution 4.0 International license (https://creativecommons.org/licenses/by/4.0).^[^ ^277^ ^]^ Copyright 2020, The Authors, published by Wiley‐VCH.

Likewise, Wang et al.^[^ ^276^ ^]^ studied CuO/TiO_2_/rGO composite and found that the synergy between CuO and rGO effectively suppresses the recombination of charge carriers and improves the surface charge transfer. It also provided more active sites for hydrogen production photocatalytic reactions, so that the maximum hydrogen evolution rate of CuO/TiO_2_‐GR was ≈20 times larger than pure P25.^[^ ^276^ ^]^ Under light illumination, the generated electrons and holes in CuO could be quickly transferred to its surface. Thus, the graphene served as an excellent electron conductor that can quickly trap the charge carriers to avoid recombination.^[^ ^268^ ^]^ Likewise, in a report presented by Huo et al.,^[^ ^269^ ^]^ graphene networks were considered as promoting material. Apart from the unique role of CuO nanoparticles known as active sites for the hydrogen production reaction, graphene networks also had a significant effect on increasing the amount of hydrogen production. The graphene networks, thanks to their porous structure, acted as a channel, and facilitated the photogenerated electrons transfer to the CuO nanoparticles.^[^ ^269^ ^]^ Consequently, the photoreduction reaction of water was improved. This reaction is also further enhanced by decreasing the CuO nanoparticles size and increasing contact surface area. It is of note that by electron injection into the CuO, the number of extra electrons shifted the CuO flat band potential to negative potentials.^[^ ^269^ ^]^ In another study, Dalapati et al.^[^ ^277^ ^]^ deposited the CuO films in the presence of poly(ethylene glycol) using the spin‐coating technique and then incorporated graphene into the CuO. The results showed that the formation of heterojunction between these two materials led to enhanced electrode stability and photoelectric properties. In this system, two different types of functional groups, including amine (–NH_2_) and carboxylic acid (–COOH) were used for graphene modification. Finally, all three electrodes (CuO, CuO:G‐NH_2_, and CuO:G‐COOH) with equal thickness were investigated. Masudy‐Panah et al. determined the optimum thickness of 500 nm for these photoelectrodes for PEC water splitting application.^[^ ^99^, ^179^ ^]^ By studying the PEC properties of synthesized electrodes under on‐off light illumination, it was found that CuO:G‐COOH composite outperforms the other electrodes in both the photocurrent density and optical stability (see **Figure** **7**a,b). After 600 s, the –COOH‐functionalized graphene‐incorporating CuO electrode retained about 70% of its initial current density. In contrast, pure CuO electrode degraded rapidly and only retained 20% of its initial current after 600 s. One reason for the photocurrent reduction in the CuO‐based composites might be the instability of this material under irradiation since CuO converts to Cu_2_O and metallic Cu over time.^[^ ^277^ ^]^ This phenomenon can be explained by using XPS analysis before and after the PEC test. As can be seen in Figure 7e–g, after PEC measurements, tiny shoulder peaks at 932.3 and 952.3 eV appeared for CuO and CuO:G‐NH_2_ films in XPS spectra, indicating the photo‐corrosion phenomenon at these electrodes.^[^ ^277^ ^]^ One of the reasons that inhibits the photo‐corrosion of CuO in the CuO:G‐COOH electrode is the electron acceptance property of the –COOH in graphene, which can capture the electron easily to prevent CuO from accessing free electrons as a result of its reduction. Another reason is that –NH_2_ has electron donor property in the CuO:G‐NH_2_ electrode, in which the reduction of CuO into Cu_2_O occurs when it supplies free electrons.^[^ ^277^ ^]^ Figure 7h,i show the photo‐corrosion of CuO electrode (conversion of CuO to Cu_2_O) before and after PEC measurement. The areas related to Cu_2_O in the morphology of the CuO electrode before and after the water splitting under the light illumination were much smaller than other electrodes under similar conditions, confirming previous results. To investigate the effect of –COOH‐functionalized graphene content on CuO:G‐COOH composite, different amounts of graphene (0.02, 0.04, and 0.06 g) were used. Based on the results of PEC characteristics (see Figure 7c,d), it was found that the CuO:G‐COOH photoelectrode with a 0.04 g of graphene content with a current density of 1.32 mA cm^−2^ at 0 V versus RHE has the best performance among all the synthesized photoelectrodes.^[^ ^277^ ^]^ Wu et al.^[^ ^254^ ^]^ synthesized Cu_2_O/CuO/rGO nanosheets by a facile one‐pot hydrothermal process. They reported that an inadequate amount of graphene not only cannot completely cover the copper oxides but there is also no significant inhibition of volume expansion in the discharge/charge process. However, using an appropriate amount of rGO (25 wt%), the charge transfer resistance of the Cu_2_O/CuO/rGO was first reduced. Next, the rGO showed excellent electrical conductivity and electrochemical properties in combination with CuO.^[^ ^254^ ^]^

**Figure 8:**
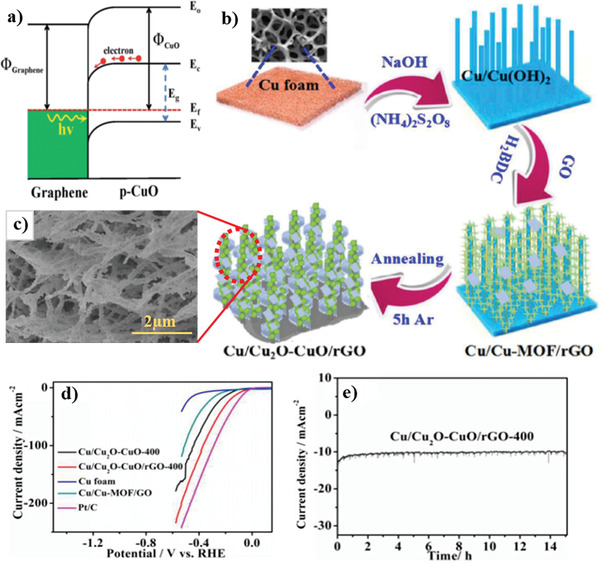
a) The energy band diagram of the graphene and CuO heterojunction under illumination. b) The general synthesis processes of the Cu/Cu_2_O–CuO/rGO. c) SEM of the Cu/Cu_2_OCuO/rGO‐400, d) Polarization curves of electrodes. e) Long‐term stability of Cu/Cu_2_O–CuO/rGO‐400. a) Reproduced with permission.^[^ ^268^ ^]^ Copyright 2020, Royal Society of Chemistry. b–e) Reproduced with permission.^[^ ^278^ ^]^ Copyright 2020, Royal Society of Chemistry.

According to the recent report by Zhang et al.,^[^ ^268^ ^]^ a Schottky junction is formed at the interface between graphene and CuO while these two materials are in contact with each other. The details of the energy band are presented in **Figure** **8**a, where the work function of graphene (φ_Graphene_ ≈ 5.0 eV) is located under the CuO level (φ_CuO_ ≈ 5.3 eV) and a small change is obvious in VB and CB edges of CuO. In this junction, the direction of the resulting internal field at the interface is determined from graphene to CuO. Therefore, electrons were migrated toward the graphene in the opposite direction of the internal field quickly. Consequently, the electron–hole separation occurred at the interface.^[^ ^268^ ^]^

Ye et al.^[^ ^278^ ^]^ prepared self‐supported bifunctional electrochemical hydrogen evolution through fabricating Cu/Cu_2_O–CuO/rGO NW arrays on the copper foam template in alkaline conditions. As shown in Figure 8b, by growing copper hydroxide NWs on the copper surface assisted in forming of Cu/Cu‐MOF/GO. Finally, by adding 1,4‐benzene dicarboxylic acid (H_2_BDC) as well as GO, as well as heat treatment at 400 °C under Ar atmosphere, the Cu/Cu_2_O–CuO/rGO was synthesized. Figure 8c indicates the 3D morphology of Cu/Cu_2_O–CuO/rGO‐400. Because of an elaborate design of the morphology and also the introduction of the highly conductive graphene, a photocurrent density of 10 mA cm^−2^ with a small overpotential of 105 mV in 1 m KOH electrolyte was obtained. However, no significant instability after 15 h was observed, indicating considerably high activity and durability of this material (see Figure 8d,e).^[^ ^278^ ^]^ The synergistic effect of graphene, combined with CuO, followed by an increase in the specific surface area, significantly improved the onset potential and current density.^[^ ^279^ ^]^

#### 5.3.2 CuO/g‐C_3_N_4_ Heterojunctions

Graphitic carbon nitride (g‐C_3_N_4_) with a bandgap energy of 2.7 eV, composed of N and C elements, regarded as one of the most popular metal‐free photocatalysts.^[^ ^280^, ^281^, ^282^, ^283^, ^284^, ^285^ ^]^ This structure has been the subject of intense research because of its ease of production, tunable electronic structures, extraordinary physical and chemical properties, and interesting electronic properties in a wide range of applications.^[^ ^286^, ^287^, ^288^, ^289^, ^290^, ^291^, ^292^, ^293^ ^]^ Besides, high electron–hole recombination is one of the significant drawbacks of pure g‐C_3_N_4_. To eliminate such obstacles, there is a greater tendency to use g‐C_3_N_4_ in composite compounds. This novel polymeric 2D layered semiconductor has shown a high potential to combine with other materials, especially metal oxides, where the formation of heterojunction structures has promoted the photocatalytic activity of the composites. For instance, g‐C_3_N_4_ facilitated the separation of the photogenerated carriers, supported the photoinduced charge‐transfer mechanisms of composites, and provided numerous active sites due to its great specific surface area after its exfoliation.^[^ ^291^, ^294^, ^295^, ^296^, ^297^, ^298^, ^299^ ^]^

**Figure 9:**
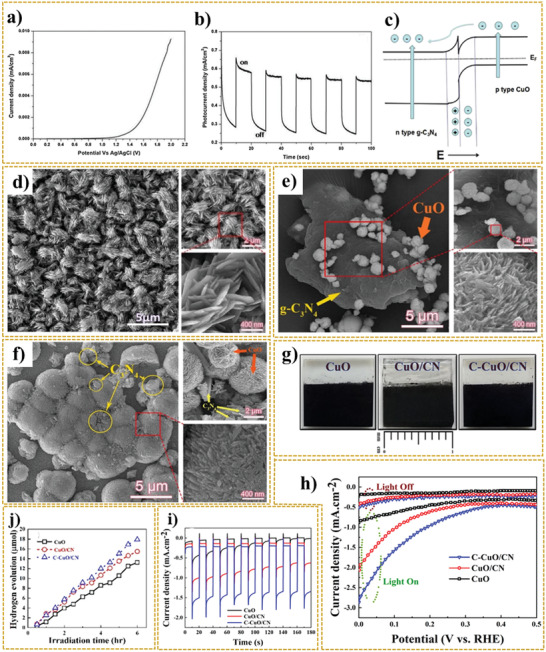
a) LSV and b) chronoamperometry measurement of CuO/g‐C_3_N_4_ nanocomposite under visible‐light irradiation. c) The mechanism of p–n heterojunction after combination. d–f) FESEM images of deposited electrodes of CuO (d), CuO/CN (e), and C–CuO/CN (f). g) Digital photographs of synthesized photocathodes. h) Linear‐sweep voltammograms curves, i) transient photocurrent responses, and j) hydrogen gas evolution of CuO, CuO/CN, and C–CuO/CN photoelectrodes. a,b) Reproduced with permission.^[^ ^300^ ^]^ Copyright 2020, Elsevier. c) Reproduced with permission.^[^ ^303^ ^]^ Copyright 2020, Elsevier. d–j) Reproduced with permission.^[^ ^213^ ^]^ Copyright 2020, Elsevier.

Recently, Ragupathi et al.^[^ ^300^ ^]^ reported the synthesis of CuO rod shape structure with an average size of 60 nm composited with g‐C_3_N_4_. This oxide improved the photocatalytic activity of water splitting under visible light irradiation. In this study, they synthesized CuO/g‐C_3_N_4_ nanocomposite using copper (II) nitrate and thiourea as cheap precursors of CuO and g‐C_3_N_4_, respectively, via a co‐deposition method in alkaline condition and subsequent heat treatment at 300 °C for 4 h. Thus, a wide range of visible light absorption for CuO/g‐C_3_N_4_ nanocomposite was observed and a narrow bandgap was estimated to be at 1.6 eV. The UV–vis absorption spectra of this nanocomposite had strong absorption under both UV and visible light irradiation. The broad peak in the wavelength range of 300–500 nm is attributed to the transfer of the lowest unoccupied molecular orbital (LUMO) to the highest occupied molecular orbital (HOMO) in the g‐C_3_N_4_, which is due to the presence of LP‐Π* and Π–Π* transitions. Also, the existence of LP‐Π* and Π–Π* transitions could cause the multi‐energy level transition.^[^ ^300^ ^]^ As shown in LSV results of the CuO/g‐C_3_N_4_ nanocomposite (see **Figure** **9**a), a photocurrent density of 0.68 mA cm^−2^ was observed at 1.2 V versus Ag/AgCl under the 100 mW cm^−2^ solar irradiation. The stability of the photocurrent performance of this nanocomposite is illustrated in Figure 9b. These results revealed the suitable stability of the CuO/g‐C_3_N_4_ nanocomposite under illumination. G‐C_3_N_4_ has a co‐catalytic role in this promising photocatalyst, which improves electron transportation performance and assists to reduce charge carrier recombination with a Schottky barrier at the junction.^[^ ^300^, ^301^ ^]^

The Mott–Schottky (M–S) diagram was plotted using the inverse square of the space charge layer capacitance versus the photocatalytic electrode potential. This analysis is used to provide information about the flat band potential and analyze the conductivity type and charge transfer of g‐C_3_N_4_ and CuO samples under constant frequency.^[^ ^301^ ^]^ The positive and negative slope of this plot matched with n‐type for g‐C_3_N_4_ and p‐type characteristics for CuO, respectively.^[^ ^301^ ^]^ Using the M–S equation, the conduction band potential of n‐type g‐C_3_N_4_ and the valence band potential of p‐type CuO were obtained at −0.86 and +1.97 V versus Ag/AgCl, respectively. Subsequently, using the bandgap energies of the semiconductors as mentioned above, the VB potential of g‐C_3_N_4_ was obtained at +1.85 eV, and the CB potential of CuO was at +0.42 eV. This electrochemical study confirmed the formation of heterojunction between g‐C_3_N_4_ and CuO.^[^ ^301^ ^]^ EIS analysis was performed to further investigate the charge transfer process in pristine g‐C_3_N_4_ and 1D/2D carbon‐CuO‐graphitic carbon nitride (C/CuO@g‐C_3_N_4_) electrodes. By studying the Nyquist plots, the hybrid electrode was able to improve effectively the separation and transfer of photogenerated electron–hole pairs and thus could enhance the electronic conductivity. The equivalent series resistance (ESR) values of C/CuO@g‐C_3_N_4_ and pristine g‐C_3_N_4_ electrodes were 6 and 8 Ω, respectively, confirming the decrement of electron transfer resistance.^[^ ^302^ ^]^ A comparison of specific surface area values between C/CuO@g‐C_3_N_4_ and g‐C_3_N_4_ indicated a 2.24‐time increase in the specific surface area of C/CuO@g‐C_3_N_4_ due to the C/CuO nanospheres structure, which increased the active sites in the hybrid electrode for electrochemical performance.^[^ ^302^ ^]^

Li et al.^[^ ^303^ ^]^ synthesized CuO/g‐C_3_N_4_ with different mass ratios of CuO/g‐C_3_N_4_ (0.5%, 1%, and 2%) by impregnation‐calcination technique to enhance the photocatalytic hydrogen evolution. The synergistic effect between CuO and g‐C_3_N_4_ resulted in the hydrogen generation rate of 937 µmol g h^−1^ after 4 h under illumination for the CuO/g‐C_3_N_4_ sample with a mass ratio of 1%, which was ≈2.17 times higher than that of pure g‐C_3_N_4_. According to the schematic of the CuO/g‐C_3_N_4_ heterojunction)see Figure 9c), the internal electric field in this system was formed from n‐type g‐C_3_N_4_ to p‐type CuO. Since this phenomenon can assist the charge carrier separation, after irradiation, the CB edge of g‐C_3_N_4_ acts as a host band for electrons, and the resultant electrons will transfer there as the active sites for hydrogen evolution. In contrast, the VB of CuO will be a host band for holes, and the generated holes at the VB of g‐C_3_N_4_ transfer to the VB of CuO.^[^ ^303^ ^]^

Hosseini H. et al.^[^ ^213^ ^]^ examined the effects of composing g‐C_3_N_4_ with cupric oxide and heat treatment of the nanocomposite electrode on the PEC performance. As shown in Figure 9d–f, they synthesized CuO micro‐flowers that consisted of intermingled ultrathin nanosheets and the hierarchical carbon‐doped CuO dandelions/g‐C_3_N_4_ (C–CuO/CN) nanocomposite. For this purpose, they used copper sulfate and urea in alkaline conditions via one‐pot microwave irradiation. Figure 9g represents the images of the synthesized photoelectrodes. C–CuO/CN nanocomposite with a unique microstructure and the bandgap of 1.3 eV exhibited a considerable photostability and superior PEC performance compared to CuO and untreated composite (CuO/CN). According to the report, the highest photocurrent density of −2.85 mA cm^−2^ at 0 V versus RHE was observed for C–CuO/CN photoelectrode under AM 1.5G illumination. This density was almost 3.3 and 1.38 times higher than that of CuO and CuO/CN, respectively (see Figure 9h). Also, as shown be in Figure 9i, C–CuO/CN photoelectrode could remarkably improve the photo‐corrosion stability of photoelectrode under visible light irradiation. As can be seen in Figure 9j, the maximum rate of hydrogen evolution of C–CuO/CN was ≈3.13 µmol h^−1^ cm^−2^. In addition, C–CuO/CN could maintain about 80% of its current density after 85 min, while CuO and CuO/CN samples kept nearly 39% and 73% of initially shown photocurrent values, respectively.

The existence of carbon nitride in the CuO/g‐C_3_N_4_ structure not only provided a suitable condition for photon absorption but also increased the effective separation of charge carriers and created appropriate pathways for the transformation of electron–hole pairs. Besides, the superior PEC performance of C–CuO/CN photocathode can be attributed to several factors such as carbon doping and the creation of defect sites, which cause the bandgap reduction. This, in turn, resulted in the improvement of light absorption, an increase in the mobility of charge carriers, and the decreased recombination rate of the photogenerated electron–hole pairs.

Moreover, as reported previously, using smaller amounts of urea only acts as fuel and can reduce particle size. Meanwhile, using the optimal amount of urea not only can control the morphology of CuO but also can lead to the formation of g‐C_3_N_4_ with the aid of CuO as the catalyst.^[^ ^213^ ^]^

**Figure 10:**
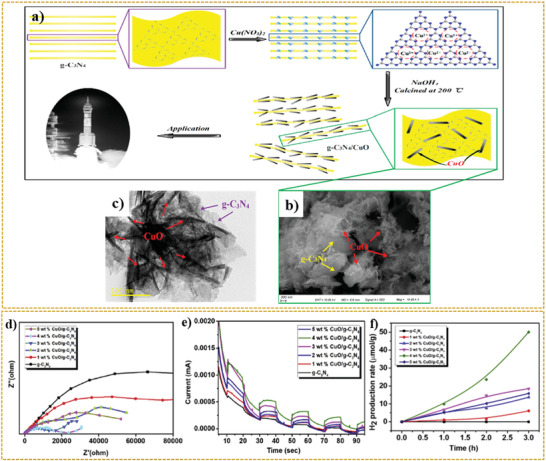
a) Schematic representation of the g‐C_3_N_4_/CuO nanocomposite formation. b) SEM and c) TEM images of g‐C_3_N_4_/CuO nanocomposite. d) Nyquist plots of EIS measurements, e) amperometric I–t curves, and f) photocatalytic H_2_ production activity for g‐C_3_N_4_ and g‐C_3_N_4_/CuO nanocomposites with various %CuO loaded photocatalysts. a–c) Reproduced under the terms of the CC‐BY Creative Commons Attribution 4.0 International license (https://creativecommons/licenses/by/4.0).^[^ ^304^ ^]^ Copyright 2020, The Authors, published by MDPI. d–f) Reproduced with permission.^[^ ^307^ ^]^ Copyright 2020, Elsevier.

Tan et al.^[^ ^304^ ^]^ decorated g‐C_3_N_4_ with CuO by a cost‐effective precipitation process by the steps shown in **Figure** **10**a. In this study, g‐C_3_N_4_ was synthesized by the conventional heating method. In this way, melamine (as a precursor of g‐C_3_N_4_) is kept at 520 °C for 2 h at a heating rate of 5 °C min^−1^ under the air atmosphere. The morphology of g‐C_3_N_4_/CuO nanocomposite is shown in Figure 10b. In the next step, the Cu(NO_3_)_2_·3H_2_O was added to the carbon nitride solution in alkaline conditions. Subsequently, the obtained powder was calcinated at 200 °C for 2 h. As a result, the CuO NRs were directly grown on g‐C_3_N_4_. The obtained NRs have a diameter of 5–10 nm and a length of 200–300 nm, confirmed in Figure 10c. Further investigation of the results showed that the CuO NRs are uniformly distributed on g‐C_3_N_4_.^[^ ^304^ ^]^

The XRD of synthesized samples confirmed the presence of the g‐C_3_N_4_ phase with characteristic peaks of 27.4° and 13.2° and the CuO phase with the major peaks of 35.6° and 38.8°, indicating the in situ formation of some CuO NRs on carbon nitride interlaminated and an increase in d spacing.

The synchronous placement of CuO NRs on the surface and the interlayer of 2D carbon nitride resulted in the CuO NRs overlapping. Hence, the formation of g‐C_3_N_4_/CuO with a unique structure was able to accelerate the charge transfer rate dramatically.^[^ ^304^ ^]^ Recent studies demonstrated that the g‐C_3_N_4_ has a strong ability for cation capturing because of the effective interaction between its negatively charged nitrogen atoms and cations.^[^ ^293^, ^304^, ^305^ ^]^ As per the prediction of researches, this ion‐dipole interaction can occur between dispersed carbon nitride and cupric ion in the solution, which paves the way for dispersing cupric ions into the carbon nitride framework.^[^ ^293^, ^304^ ^]^

Shi et al.^[^ ^306^ ^]^ synthesized heterostructure g‐C_3_N_4_/CuO*_x_* nanocomposites with a high specific surface area with different amounts of g‐C_3_N_4_ by a mixed solvent‐thermal method. For this purpose, the surface of CuO*_x_* composites was completely covered using appropriate amounts of ultrathin exfoliated g‐C_3_N_4_. According to the classification of Brunauer–Deming–Deming–Teller (BDDT), the nitrogen adsorption–desorption isotherms plots of synthesized samples are type IV and the results of high adsorption at relative pressures (*P*/*P*_0_) were close to 1.0, indicating the large mesopores and slit pores.^[^ ^306^ ^]^ It was found that by increasing the amount of carbon nitride, the specific surface area is also increased. Undoubtedly, by increasing the effective specific surface area, the active sites also expanded and eventually improved the photocatalytic properties. Nevertheless, the 0.5g‐C_3_N_4_/CuO*_x_* sample displayed significant inhibition of photo‐generated electron–hole pairs, in which a mixed solvothermal process can be a potential reason for the difference of the maximum emission peak between the highest and lowest intensity. Based on the transient photocurrent responses, the current density of the electrodes was measured under visible light irradiation. As expected, the g‐C_3_N_4_/CuO*_x_* nanocomposite performed better in the same condition, and its photocurrent response intensity was higher than that of CuO*_x_* and bulk g‐C_3_N_4_.^[^ ^306^ ^]^

Karthik et al.^[^ ^307^ ^]^ also synthesized CuO/g‐C_3_N_4_ heterojunction through a wet impregnation process. The results showed a better photocurrent response of CuO loaded g‐C_3_N_4_ than pure g‐C_3_N_4_ because of its ability to generate electron–hole pairs and better charge carrier separation. As can be seen in Figure 10d, the smaller arc radius of CuO/g‐C_3_N_4_ nanocomposite confirmed this content compared to the pure g‐C_3_N_4_ in Nyquist plots, which revealed the preferred transfer rate of the photogenerated charges at the electrode/electrolyte interface. Moreover, in this study, the Mott–Schottky of g‐C_3_N_4_ and CuO/g‐C_3_N_4_ photocatalyst was investigated, which provided information about the flat band potentials estimated at −1.3 for g‐C_3_N_4_ and −0.95 V versus NHE for CuO/g‐C_3_N_4_.^[^ ^307^, ^308^ ^]^ Thus, tuning the conduction flat band potential to more positive values indicated the formation of a p–n heterojunction between p‐type CuO and n‐type g‐C_3_N_4_ photocatalysts.

As depicted in Figure 10e, loading various percentages of CuO with g‐C_3_N_4_ improved both charge carrier separation and charge carrier production. As shown in Figure 10f, the maximum hydrogen generation rate after 3 h is related to the sample of 4 wt% CuO/g‐C_3_N_4_ with 50.1 µmol g^−1^. The charge transfer mechanism of the samples was examined under visible light irradiation. After the excitation, according to the position difference of CBs of photocatalysts, the photogenerated electrons can be transferred to the CB of CuO. It has been reported, the Fermi level shifts toward the negative potential due to excessive electron accumulation on the CuO surface. Hence, the abovementioned characteristics enable the excellent spatial separation of charge carriers at the interface.^[^ ^307^, ^309^ ^]^ Besides, due to the increase of CuO percentage, the d–d transitions of Cu (II) were also increased, leading to the extension of the UV–vis range. Thus, the photocatalytic performance of the CuO‐g‐C_3_N_4_ nanocomposite was improved. Another plausible charge transfer mechanism for g‐C_3_N_4_/CuO nanocomposite was discussed by Hong et al. The VB and CB of CuO with the value of +1.14 V versus SHE and −0.46 V versus SHE, respectively, are located between the CB and VB flat bands potential of g‐C_3_N_4_.^[^ ^310^ ^]^

#### 5.3.3 CuO/Dichalcogenides Heterojunctions

2D transition metal dichalcogenides (2D TMDs) are among the essential groups in 2D family materials. The formula of this class of semiconducting materials is MX_2_, where M denotes the transition metals such as Ti, Fe, Ni, Mo, and W and X refers to chalcogens such as S, Se, and Te.^[^ ^311^, ^312^, ^313^, ^314^, ^315^, ^316^, ^317^, ^318^ ^]^

Recently, TMDs with their unique crystal structures, superior properties, and exotic functionalities have drawn intense attention in applications for piezoelectric devices,^[^ ^319^, ^320^, ^321^ ^]^ batteries,^[^ ^322^, ^323^, ^324^ ^]^ electronic devices,^[^ ^325^, ^326^ ^]^ photocatalysis,^[^ ^327^, ^328^, ^329^, ^330^, ^331^ ^]^ biomedicine,^[^ ^332^, ^333^, ^334^ ^]^ and PEC sensing.^[^ ^335^, ^336^ ^]^ In this regard, 2D TMDs can play an important role in energy production in the future. These materials, because of their remarkable energy bandgap, the facilitation of charge carrier transport, the ability to increase the active sites, tuning the phase, and electronic structure are suitable candidates for PEC applications.^[^ ^337^, ^338^ ^]^ So far, various studies have been conducted on the properties of integrating TMDs with different compounds, including graphene materials, metal oxides, g‐C_3_N_4_, and CdS^[^ ^339^, ^340^, ^341^, ^342^ ^]^ to increase the photocatalytic properties of water splitting. Nevertheless, to the best of our knowledge, only a few reports have been published about TMDs composited with CuO for PEC water splitting.

**Figure 11:**
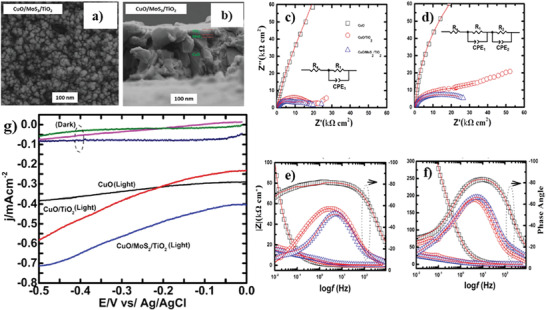
a) Cross‐sectional and b) FESEM image of CuO/MoS_2_/TiO_2_ thin film. c,d) Nyquist plots of ITO/CuO, ITO/CuO/TiO_2_, and ITO/CuO/MoS_2_/TiO_2_ films with their equivalent circuits in light (c) and dark (d) conditions. e,f) Bode plots of synthesized electrodes in the light (e) and dark (f) conditions. g) Current–voltage measurements of ITO/CuO, ITO/CuO/TiO_2_, and ITO/CuO/MoS_2_/TiO_2_ films versus Ag/AgCl in the 0.5 M Na_2_SO_4_ solutions in light and dark conditions. a–g) Reproduced with permission.^[^ ^343^ ^]^ Copyright 2020, Springer Nature.

Mahmood et al.^[^ ^343^ ^]^ investigated the effect of MoS_2_ as the intermediate layer between CuO and TiO_2_ for PEC hydrogen production. First, the CuO film was prepared through the galvanostatic deposition method in alkaline conditions with a continuous current density of −0.3 mA cm^−2^ from 300 to 1200 s. Then, the deposited film was annealed at 550 °C for 2 h to convert into CuO. Afterward, a certain amount of dispersed MoS_2_ and TiO_2_ mixture was spin‐coated on the surface of the CuO films under a specific condition. For better electrical contact, the obtained film was annealed at 300 °C for 1 h. **Figure** **11**a,b shows the morphology and cross‐sectional images of CuO and CuO/MoS_2_/TiO_2_ films in higher magnification, where the film thicknesses were calculated at 0.8 to 1 µm.

As can be seen in Figure 11c,d, the electrochemical impedance analysis of electrodes was performed in both dark and light conditions. Due to the large radius of the ITO/CuO electrode (magnitude of *R*_p_) in the Nyquist plots, it is evident that the charge transfer resistance of the electrolyte–electrode interface for this system is much higher than those of other deposited electrodes. The value of *R*_p_ was 729.170 Ω for ITO/CuO and 18.490 Ω for ITO/CuO/TiO_2_. With the introduction of MoS_2_ into the CuO–TiO_2_ composite, this value decreased to 17.388 Ω for ITO/CuO/MoS_2_/TiO_2_. This reduction in *R*_p_ indicated the improvement of PEC response. The MoS_2_, as a driving force, could increase the charge transfer kinetics in the CuO–TiO_2_ composite system by creating an efficient interlayer. In this study, to further evaluate the behavior of the transferred charge, the plots of |Z| versus log frequency were investigated (Figure 11e,f) where |Z| refers to the complex number of the impedance, and it is defined as Equation (1):

(1) $$\begin{array}{c} \left|Z\right|={\left[{\left(Z\prime \right)}^{2}+{\left(Z\prime \prime \right)}^{2}\right]}^{1/2} \end{array}$$

where Z′ and Z″ are the real and imaginary components, respectively. According to this plot, a descending trend is seen for |Z|. Moreover, the Bode plots (phase angle versus log frequency) exhibited the particular maximum for the electrodes, where the maximum for ITO/CuO, ITO/CuO/TiO_2_, and ITO/CuO/MoS_2_/TiO_2_ electrodes corresponds to a phase angle of −80°, −60°, and −50°, respectively. Depending on where the maximum peak is located, the cause of its formation is divided into two categories. If it is located at a low frequency, its configuration could be attributed to the electron diffusion in electrolytes. In contrast, if it is located at a high frequency, it could be associated with the resistance in the semiconductor. So, the ITO/CuO/MoS_2_/TiO_2_ electrode displayed less resistance to charge mobility, supporting the results of EIS analysis. Such properties prove the excellent performance of this electrode in the PEC system.^[^ ^343^ ^]^ Figure 11g represents the photocurrent density of −0.73 mA cm^−2^ for the ITO/CuO/MoS_2_/TiO_2_ electrode under standard solar light illumination.

The authors believe that MoS_2_ in CuO/TiO_2_ composite system not only can act as an electron sink to prevent electron–hole recombination but also can provide conditions for charge transfer in the interface and significantly increase active sites for photocatalytic reactions center.^[^ ^343^ ^]^

A concise summary of the photocurrent density of CuO/2D composites is given in **Table** **4**. All reported photocurrents in this table are in a laboratory scale and thus more efforts should be done to develop this category of photocatalysts in large scale.

**Table 4:** Summary photocurrents of CuO/2D nanocomposites electrodes by different synthesis procedures for PEC water splitting

Sn.	Type of photocathode	Fabrication process	Photocurrent density^a)^	Ref.
1	Cu/Cu_2_O–CuO/rGO	Simple calcination of Cu/Cu‐MOF/GO	10 mA cm^−2^ at overpotential of 105 mV versus RHE	^[^ ^278^ ^]^
2	CuO–Cu_2_O–Cu nanorod‐decorated reduced graphene oxide	Hydrothermal	0.23 µA at 0 V versus Ag/AgCl	^[^ ^275^ ^]^
3	–COOH‐functionalized graphene into the CuO film (CuO:G‐COOH)	Sol–gel method	−1.32 mA cm^−2^ at 0 V versus RHE	^[^ ^277^ ^]^
4	–COOH‐functionalized graphene into the CuO film (CuO:G‐COOH) with TiO_2_ protecting layer (CuO:G‐COOH)–TiO_2_	Sol–gel method	−1.75 mA cm^−2^ at 0 V versus RHE	^[^ ^277^ ^]^
5	–COOH‐functionalized graphene into the CuO film (CuO:G‐COOH) with TiO_2_ protecting layer and Au–Pd co‐catalyst nanostructures (CuO:G COOH)–TiO_2_–AuPd	Sol–gel method and RF sputtering	−2.5 mA cm^−2^ at 0 V versus RHE	^[^ ^277^ ^]^
6	CuO/g‐C_3_N_4_	Co‐precipitation	−0.68 mA cm^−2^ at 1.2 V versus Ag/AgCl	^[^ ^300^ ^]^
7	0.5 g C_3_N_4_/CuO*_x_* (CN_5_/CuO*_x_*)	Mixed solvent‐thermal method	≈1.8 mA cm^−2^	^[^ ^306^ ^]^
8	CuO/MoS_2_/TiO_2_	Galvanostatic deposition method	−0.73 mA cm^−2^ −0.5 V versus Ag/AgCl	^[^ ^343^ ^]^
9	CuO/MoS_2_	Potentiostatic deposition + thermal treatment	−1.64 mA cm^−2^ −0.55 V versus Ag/AgCl	^[^ ^127^ ^]^
10	CuO/g‐C_3_N_4_	One‐pot microwave synthesis	−2.06 mA cm^−2^ at 0 V versus RHE	^[^ ^213^ ^]^
11	Carbon‐doped CuO/g‐C_3_N_4_	One‐pot microwave synthesis + heat treatment	−2.85 mA cm^−2^ at 0 V versus RHE	^[^ ^213^ ^]^

^a)^ Some values are estimated based on the data/graphs presented in the literature.

Titanium disulfide (TiS_2_) with an energy bandgap in the range of 0.05–2.5 eV,^[^ ^344^, ^345^ ^]^ and S–Ti–S structure is one of the unique semiconductors in 2D TMDCs. The intralayer sulfur (S)–titanium (Ti) bonds are covalent whereas weak van der Waals interactions occur between TiS_2_ nanosheets. It has been reported that by increasing the weight percent of CuO in the CuO/TiS_2_ composite, the PL emission peaks of composite samples significantly decreased compared to the pure TiS_2_, indicating the suppression of recombination of charge carriers in CuO/TiS_2_ composite. Moreover, due to the formation of a junction between CuO and TiS_2_ semiconductors, the bandgap of TiS_2_ was decreased from 2.20 to 1.85 eV for 50% CuO‐TiS_2_ nanocomposite. The mentioned factors have made this nanocomposite usable in photocatalytic applications.^[^ ^344^ ^]^

**Figure 12:**
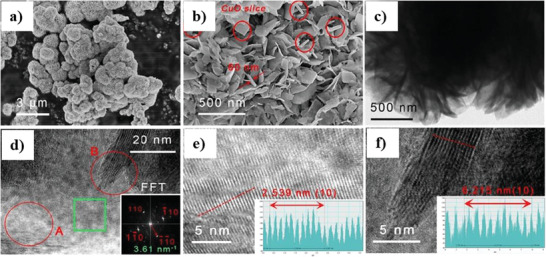
a,b) SEM images of the MoS_2_@CuO hetero‐nanoflowers at different magnifications. c) TEM image of MoS_2_@CuO nanocomposite. d–f) HR‐TEM image of a MoS_2_@CuO nanocomposite with FFT pattern. The images in (e,f) belong to zone A and B, respectively. a–f) Reproduced with permission.^[^ ^346^ ^]^ Copyright 2020, Royal Society of Chemistry.

For the first time, Li et al.^[^ ^346^ ^]^ synthesized a novel MoS_2_@CuO heterogeneous structure with nanoflower morphology via a two‐step hydrothermal route in an acidic condition. As confirmed by the SEM images in **Figure** **12**a,b, there are CuO slice‐like formations with thickness around 60 nm into the part of MoS_2_ petals. Moreover, the TEM images in Figure 12c–f, confirmed the crystalline structure and the strong chemical coupling between MoS_2_ and CuO, which indicated a heterojunction structure between these two semiconductors. In the systems containing heterojunction between the two semiconductors, due to some obstacles such as improper band offset of the interface, the transfer of electrons is delayed such that it can reduce the efficiency of photocatalytic properties. Several researches have been done in this field, including the use of modeling and simulation methods for investigating photocatalytic systems to increase the efficiency of these systems under sunlight irradiation.^[^ ^347^, ^348^ ^]^ Recognizing the obstacles and knowing how electrons are transmitted in such systems will improve their optical and photocatalytic applications. In this regard, Li et al.^[^ ^346^ ^]^ tried to extract information around the flat bond potentials between MoS_2_ and CuO (valence band offset (VBO) and conduction band offset (CBO)) using the results of XPS analysis and Kraunt's method.

Based on the results, they found that the heterojunction structure formed between CuO and MoS_2_ is type‐II band alignment. Also, the small VBO/CBO ratio obtained in this study not only facilitates the separation of the charge carriers in the interface but also promotes better migration of electrons to the surface.^[^ ^346^ ^]^

A remarkable enhancement in the photocatalytic activity of MoS_2_/CuO nanocomposite compared to CuO and MoS_2_ can be attributed to the improvement of these factors, which in turn improved the growth of single surface photovoltage (2.5 times versus MoS_2_) and increased specific surface area from 20.06 m^2^ g^−1^ for MoS_2_ to 23.5 m^2^ g^−1^ for MoS_2_@CuO heterogeneous structure nanoflowers.^[^ ^346^ ^]^

**Figure 13:**
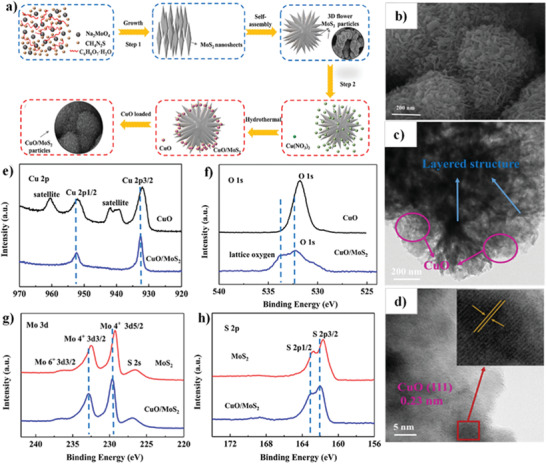
a) Schematic illustration of the synthesis process of CuO/MoS_2_ composite via the hydrothermal method. b–d) SEM and TEM images of 50% CuO/MoS_2_ sample. e,f) High‐resolution XPS analytic results of pure CuO and 50% CuO/MoS_2_ samples. g,h) High‐resolution XPS analytic results of pure MoS_2_ and 50% CuO/MoS_2_ samples. a–h) Reproduced with permission.^[^ ^350^ ^]^ Copyright 2020, Elsevier.

Sharma et al.^[^ ^349^ ^]^ studied the effect of making the electrical connection was determined on the band diagram of *n*‐MoS_2_/p CuO heterojunction. Hu et al.^[^ ^350^ ^]^ proposed a generally hydrothermal method (see **Figure** **13**a) for preparing CuO/MoS_2_ composites. The obtained SEM and TEM in Figure 13b–d shows the wrinkles on the outer layer of MoS_2_ structure and distribution of CuO nanoparticles on the surface of MoS_2_. This unique nanostructure showed a specific surface area higher than that of pure CuO and MoS_2_. Accordingly, the surface area of the 50%‐CuO/MoS_2_ sample was 23.1 m^2^ g^−1^, which was 11.5 and 1.5 times larger than that of CuO and MoS_2_, respectively. This magnitude of the specific surface area of the composite sample can be attributed to the vital role of MoS_2_ as a supporter, which led to the successful distribution of CuO nanoparticles on the surface of MoS_2_. In this study, they used XPS to examine the interaction between MoS_2_ and CuO in MoS_2_/CuO nanocomposite and the chemical behavior of the elements in this nanocomposite. Hence, considering the position of the core level peaks before and after composition in Figure 13e,f, the formation of heterojunction was proved. The position of Cu 2p_3/2_, Cu 2p_1/2_, and O 1s peaks was 932.67, 952.46, and 532.0 eV, respectively, in the CuO sample. Meanwhile, after the formation of nanocomposite between CuO and MoS_2_, the position of these peaks shifted to the higher binding energy position. In addition, no trace of satellite peaks was observed in the composite sample. Likewise, as shown in Figure 13g,h, after the formation of the composition, the same trend happened for the core levels of MoS_2_ (Mo^4+^ 3d_5/2_, Mo^4+^ 3d_3/2_, Mo^6+^ 3d_3/2_, S 2s, S 2p_1/2_, and S 2p_3/2_), which shifted to the higher binding energy position. The results of these studies showed a strong interaction between the two semiconductors.^[^ ^350^ ^]^

## 6 Summary and Perspectives

In the present study, the development of CuO‐based photoelectrodes with a promising performance in PEC water splitting is comprehensively discussed. By investigating various morphologies and synthesis methods of CuO photoelectrode, it was conceived that these two parameters play a critical role in the obtained photocurrent density and consequently control the PEC water splitting efficiency. Moreover, summarizing the details of the available and essential parameters, it is confirmed that for the fabrication of highly photoactive as well as photostable material, enhanced surface area, tuned crystallinity, the lower recombination rate of charge carriers, and appropriate electrolyte are the key metrics. Thus, the synthesis of some specific morphologies increases the specific surface area and results in the effective separation of electron–hole pairs. Also, modifying the CuO crystal structure revealed a significant influence on the enhancement of the photocurrent density and photostability. The optimized morphology and CuO composition resulted by using the CuO/ZnO‐NW photoelectrode exhibited high photocurrent density of −8.1 mA cm^−2^ at 0 V versus RHE, which was fabricated by dip‐coating of CuO films in a solution having well‐dispersed zinc oxide nanoparticles. Furthermore, the synergetic photocatalytic effect of CuO and 2D materials heterojunctions such as CuO/2D carbon material, CuO/g‐C_3_N_4_, and CuO/dichalcogenides (TiO_2_/MoS_2_) was found to be more effective and offered better charge mobility at the proximity to the junction of the electrode/electrolyte within the nanostructure. Therefore, the heterojunctions are known to be responsible for decreasing the recombination rate of the photogenerated charge carriers. Moreover, the complex role of doping agents is discussed as they can raise the photostability of prepared photocathodes in some cases and reduce the photocurrent density in some others. Since photoinduced decomposition of CuO and Cu_2_O photoelectrodes result in photo‐corrosion, thus surface treatments or a thin layer of protective coatings can be an effective strategy to enhance the photostability. In this regard, carbon‐doped CuO dandelions/g‐C_3_N_4_ photoelectrodes exhibited a considerable photostability and superior PEC performance, which retained ≈80% of its current density after 85 min, with high photocurrent density of −2.85 mA cm^−2^ at 0 V versus RHE. Although many efforts have been consecrated to improve the PEC water splitting by using the CuO‐based electrodes for hydrogen evolution, more research is still required to enhance their photocatalysis performance. Besides, overcoming the manufacturing cost and scale‐up challenges of the modified CuO photoanodes is of great importance for future industrial applications. In conclusion, the current investigation illustrated a great potential for the continuation of research to enhance CuO‐based photoelectrodes for applications in PEC water splitting.

## Conflict of Interest

The authors declare no conflict of interest.
